# Supplementary material for: Heterologous expression reveals the biosynthesis of the antibiotic pleuromutilin and generates bioactive semi-synthetic derivatives
Source: Nat Commun. 2017 Nov 28;8:1831. doi: 10.1038/s41467-017-01659-1 (PMC5705593; doi:10.1038/s41467-017-01659-1)
Supplement: Supplementary file 1 — Supplementary Information [file 41467_2017_1659_MOESM1_ESM.pdf]

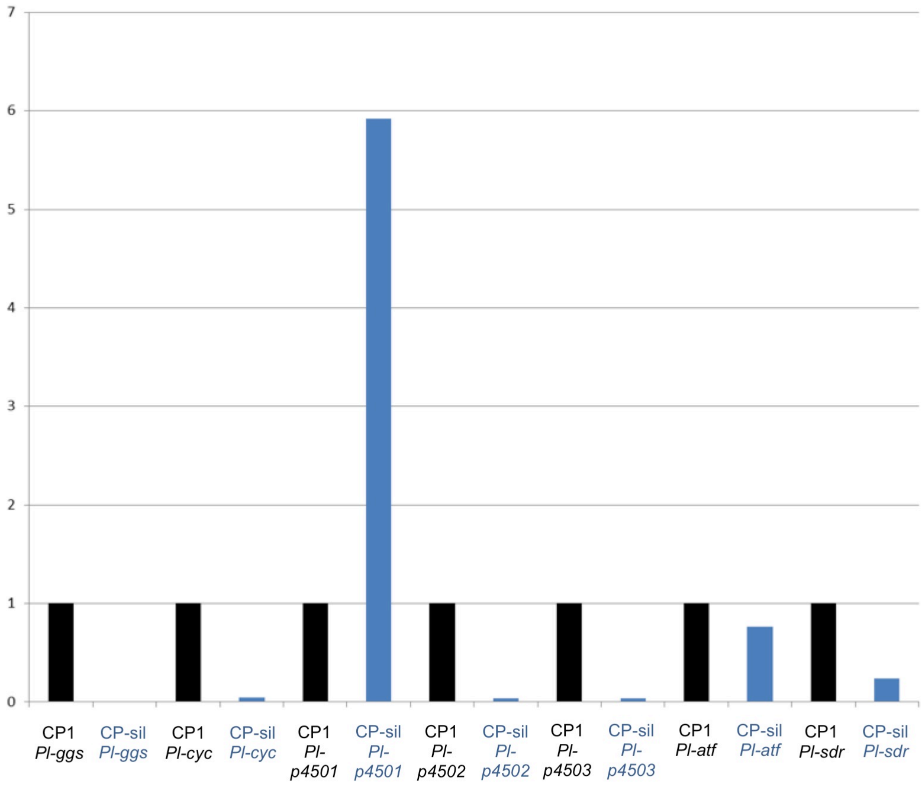

Supplementary Figure 1. Transcription analyses on *C. passeckerianus*. Quantitative reverse transcription PCR (RT-qPCR) data presented as a chart for abundance of pleuromutilin-related gene transcripts in *C. passeckerianus Pl-atf*-silenced line (labelled as CP-sil, histograms in blue) in comparison with the expression of the same genes in *C. passeckerianus* wild type (labelled as CP1, histograms in black). A clear reduction in the accumulation of detectable transcript is observed in strain CP-sil for all but one gene of the pleuromutilin cluster, *Pl-p450-1*. The nearly six-fold increase in expression of this gene was unexpected. Detectable levels of gene *Pl-atf* were also higher than expected, which may be due to detection in RT-qPCR of the antisense transcript used for the silencing experiment. Besides, the two genes *Pl-ggs* and *Pl-cyc*, supposed to code for the first two enzymes involved in the pleuromutilin pathway, appeared to be considerably downregulated compared to CP1. This explains the lack of accumulation of intermediate compounds from strain CP-sil.

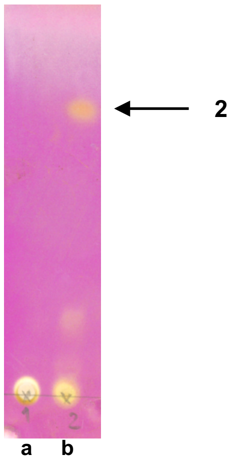

Supplementary Figure 2. TLC of *A. oryzae* transformant GC extract. TLC of crude extracts from the control strain *A. oryzae* NSAR1 (**a**), and the transformant strain GC (**b**), which is showing production of **2**. The solvent system used was petroleum spirit-ethyl acetate (9:1).

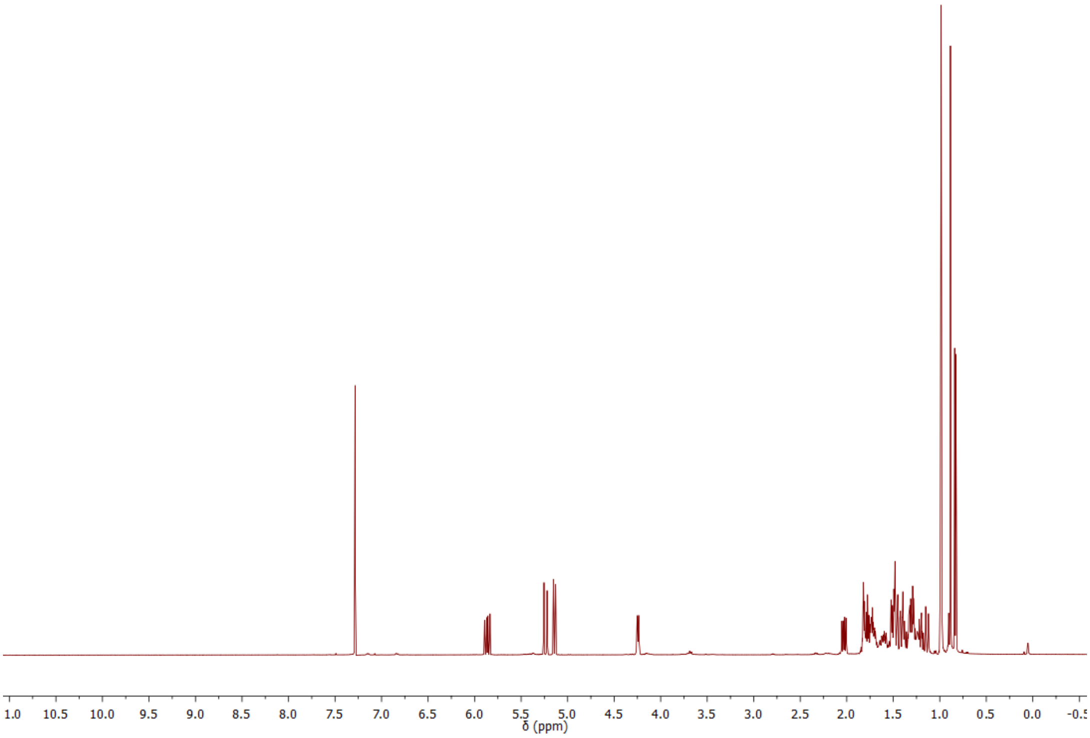

Supplementary Figure 3. ^{1}H-NMR spectrum of **2** in $CDCl_3$ (500 MHz).

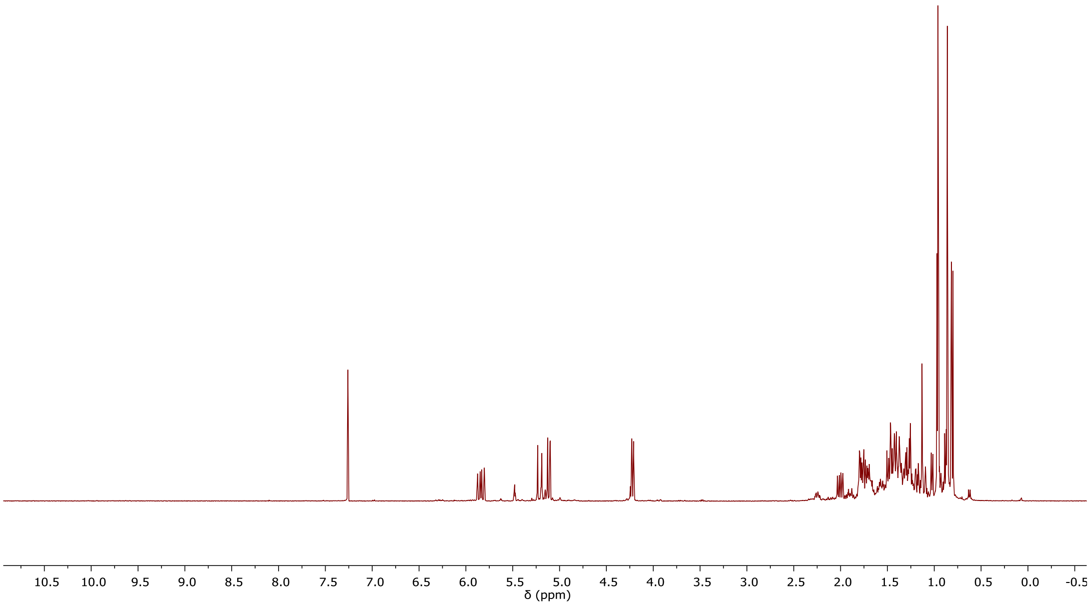

Supplementary Figure 4. ^{1}H-NMR spectrum of synthetic **2** in $CDCl_3$ (500 MHz).

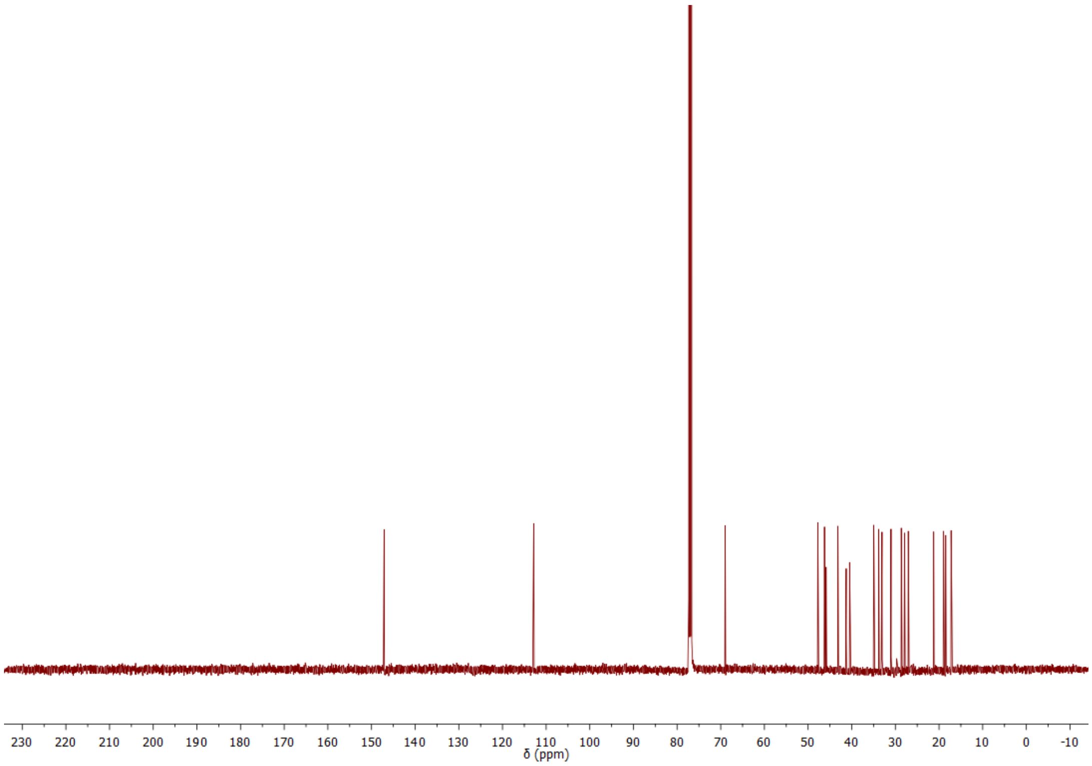

Supplementary Figure 5. ^{13}C-NMR spectrum of **2** in $CDCl_3$ (125 MHz).

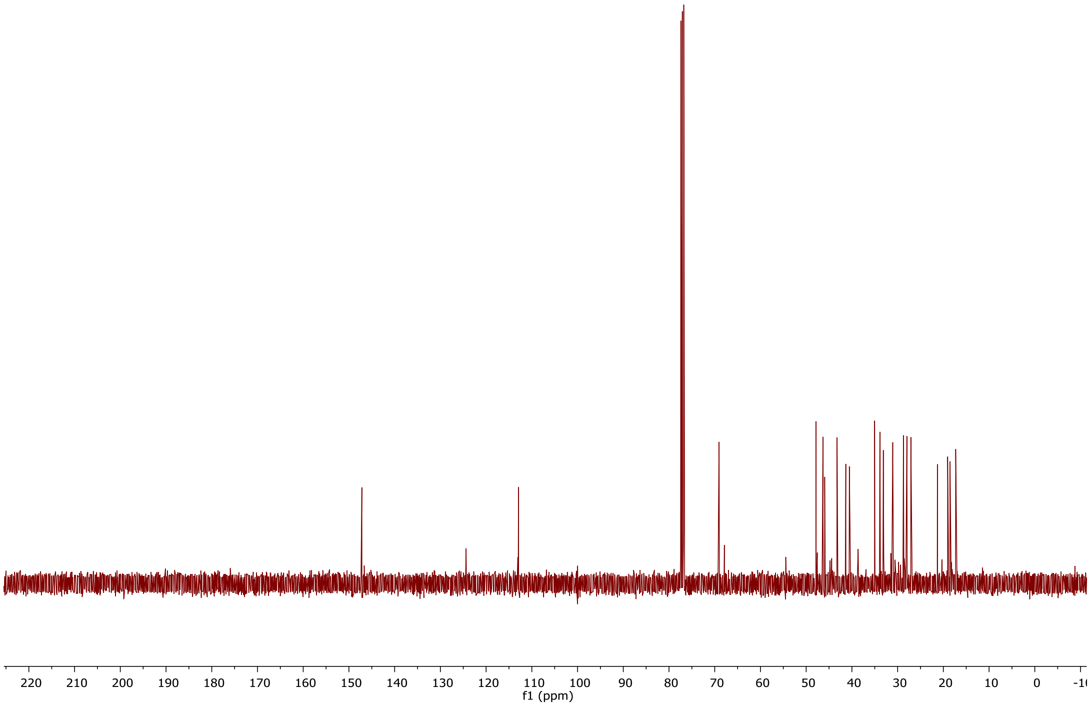

Supplementary Figure 6. ^{13}C-NMR spectrum of synthetic **2** in $CDCl_3$ (125 MHz).

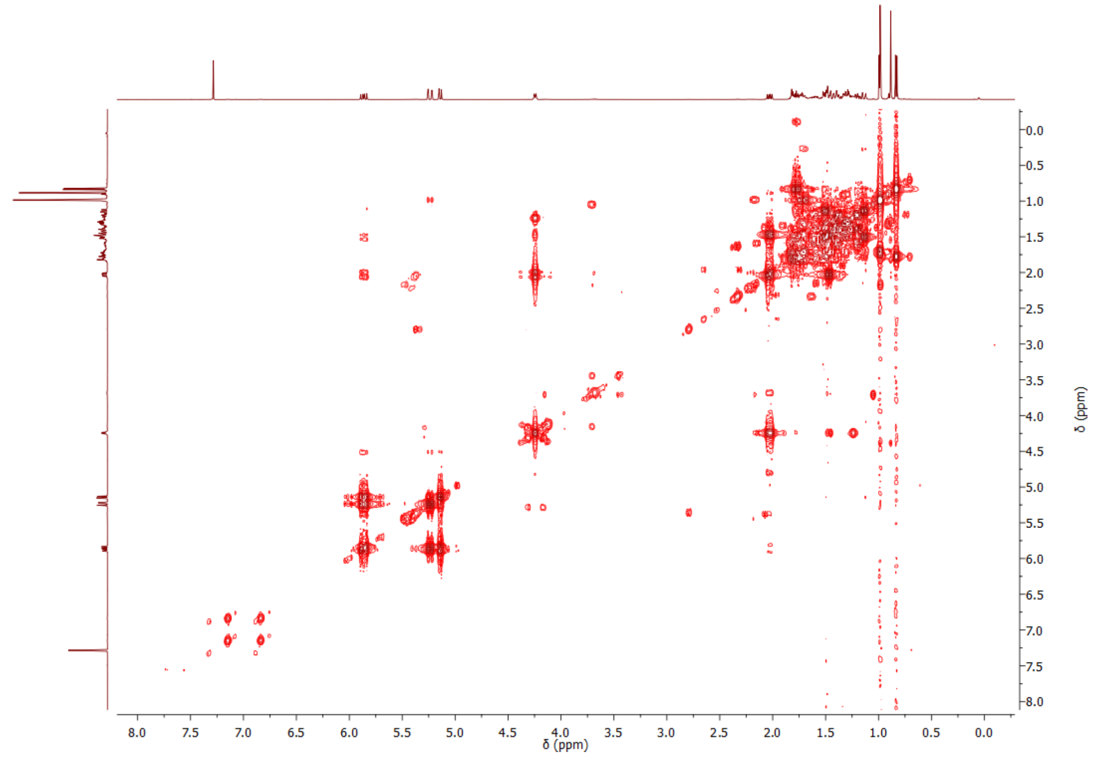

Supplementary Figure 7. COSY spectrum of **2** in $CDCl_3$ (500 MHz).

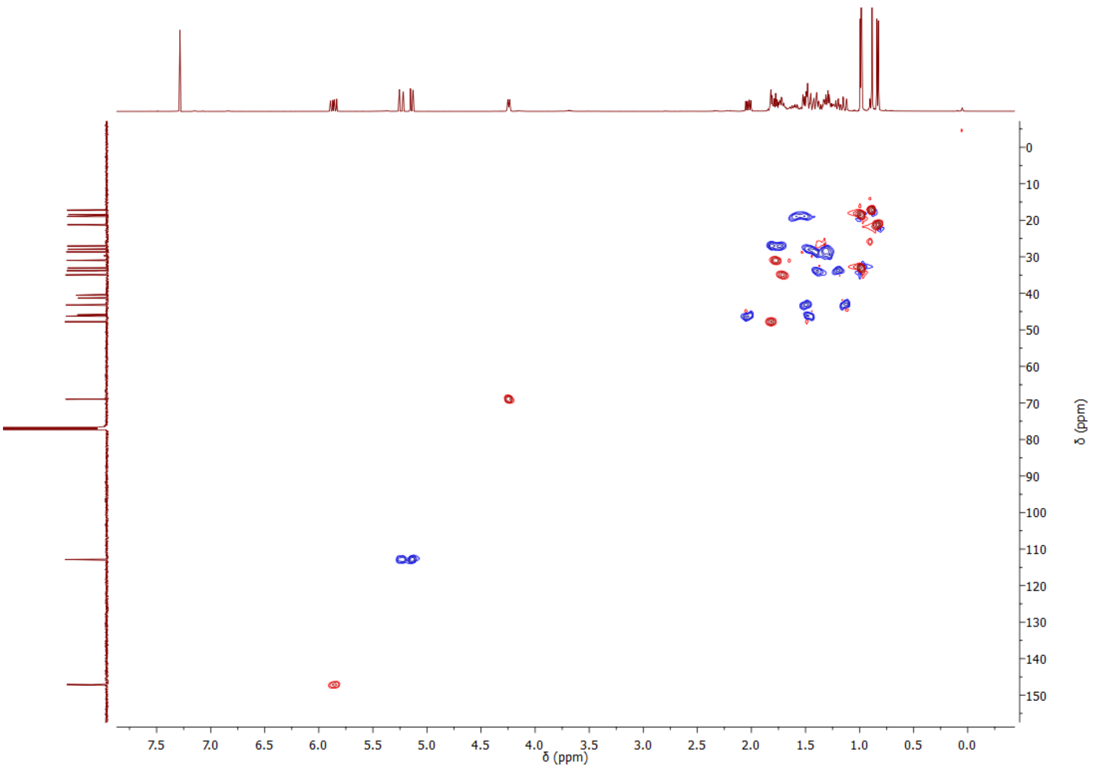

Supplementary Figure 8. HSQC spectrum of **2** in $CDCl_3$ (500 MHz).

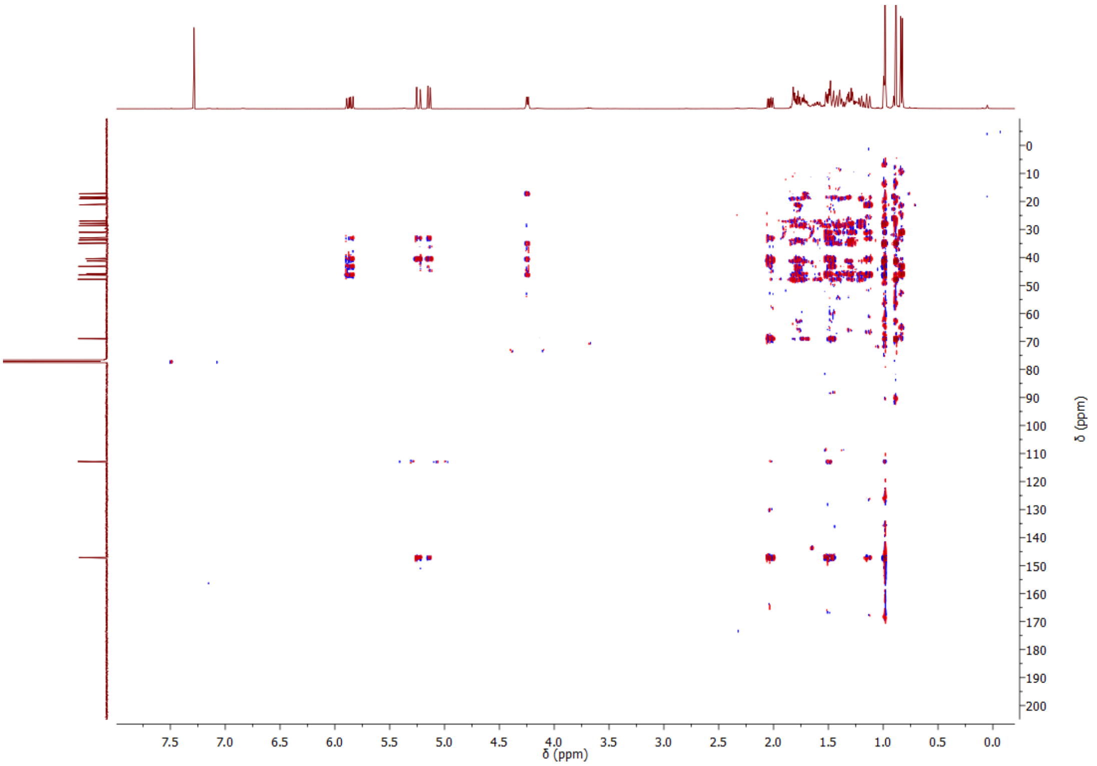

Supplementary Figure 9. HMBC spectrum of **2** in $CDCl_3$ (500 MHz).

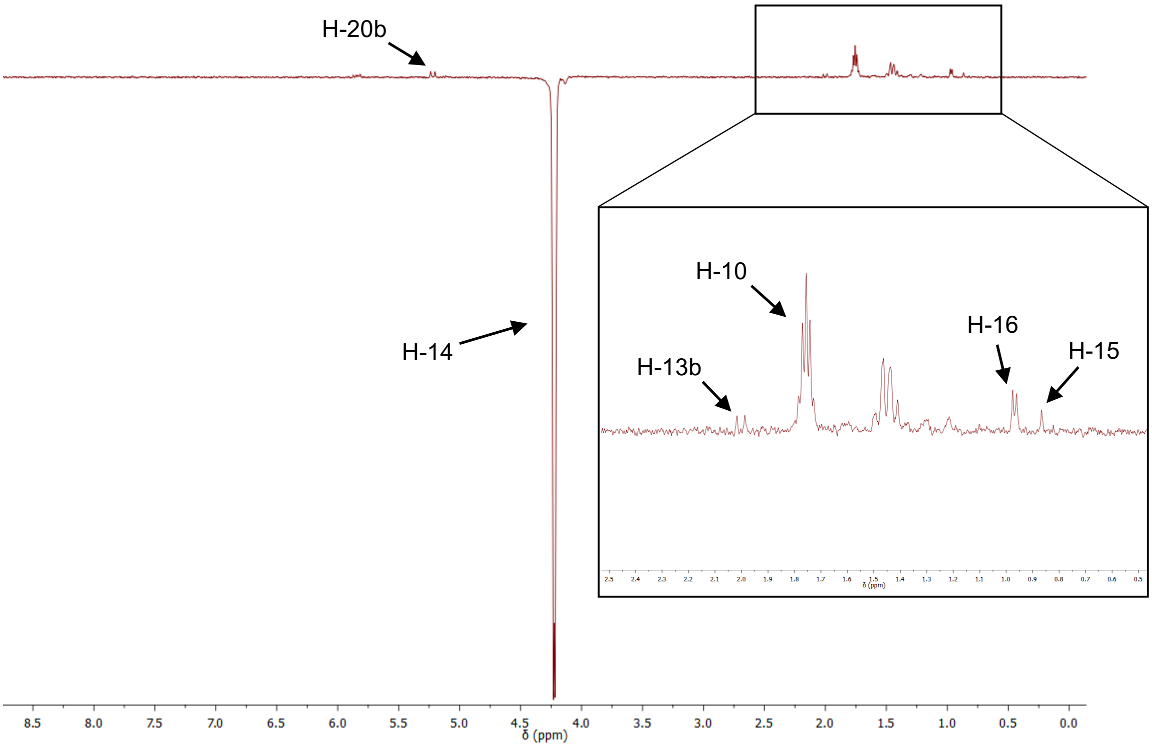

Supplementary Figure 10. Selective 1D-NOESY of **2** in $CDCl_3$. The proton H-14 was selectively irradiated (4.22 ppm). The spectrum showed enhanced proton signals for H-10, H-13b, H-15, H-16 and H-20b.

Supplementary Table 1. NMR data assignment of **2** in $CDCl_3$. CIMS was used to determine the molecular formula of **2** (CIMS: *m/z* 291.2688 $[M+H]^+$, calculated for $C_{20}H_{35}O$: 291.2678 $\Delta = 1.0$ mmu).

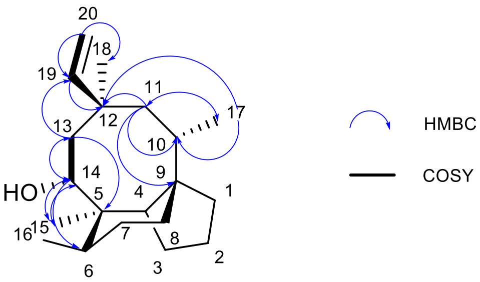

			Experimental data for **2**					Reference*
#	$^{13}C(\delta)$	HSQC	$^{1}H(\delta)$	*J* Coupling (Hz)	Protons	HMBC	COSY	$^{13}C(\delta)$
1	28.8	CH_2	1.24-1.36	m	1			28.7
			1.37-1.52	m	1			
2	19.1	CH_2	1.52-1.67	m	1			19.0
			1.52-1.67	m	1			
3	27.2	CH_2	1.68-1.75	m	1			27.0
			1.75-1.84	m	1			
4	47.9	CH	1.78-1.81	m	1			47.7
5	41.4							41.2
6	35.1	CH	1.65-1.72	m	1			35.0
7	28.1	CH_2	1.28-1.33	m	1			27.9
			1.39-1.43	m	1			
8	34.0	CH_2	1.14-1.28	m	1			33.8
			1.34-1.39	m	1			
9	40.6							40.4
10	31.1	CH	1.72-1.78	m	1			30.9
11	43.3	CH_2	1.08-1.13	m	1	C-9, C-10, C-12, C-17, C-19		43.1
			1.48-1.50	m	1			
12	46.0							45.8
13	46.4	CH_2	1.43-1.50	m	1			46.3
			2.01	dd, 15.1, 7.9	1	C-5, C-14, C-18, C-19		
14	69.1	CH	4.22	d, 7.9	1	C-6, C-9, C-13, C-15	H-13b	68.8
15	17.4	CH_3	0.86	s	3	C-4, C-5, C-6, C-14		17.2
16	18.6	CH_3	0.96	d, 7.0	3			18.5
17	21.4	CH_3	0.81	d, 6.9	3	C-10, C-11, C-12		21.2
18	33.2	CH_3	0.96	s	3			33.0
19	147.3	CH	5.84	dd, 17.8, 11.0	1	C-9, C-11, C-12	H-20a, H-20b	147.0
20	113.0	CH_2	5.12	dd, 11.0, 1.2	1	C-9, C-18, C-19	H-19	112.9
			5.21	dd, 17.8, 1.2	1	C-9, C-18, C-19	H-19	

*Reported ^{13}C-NMR chemical shifts for **2** from Hasler[1]

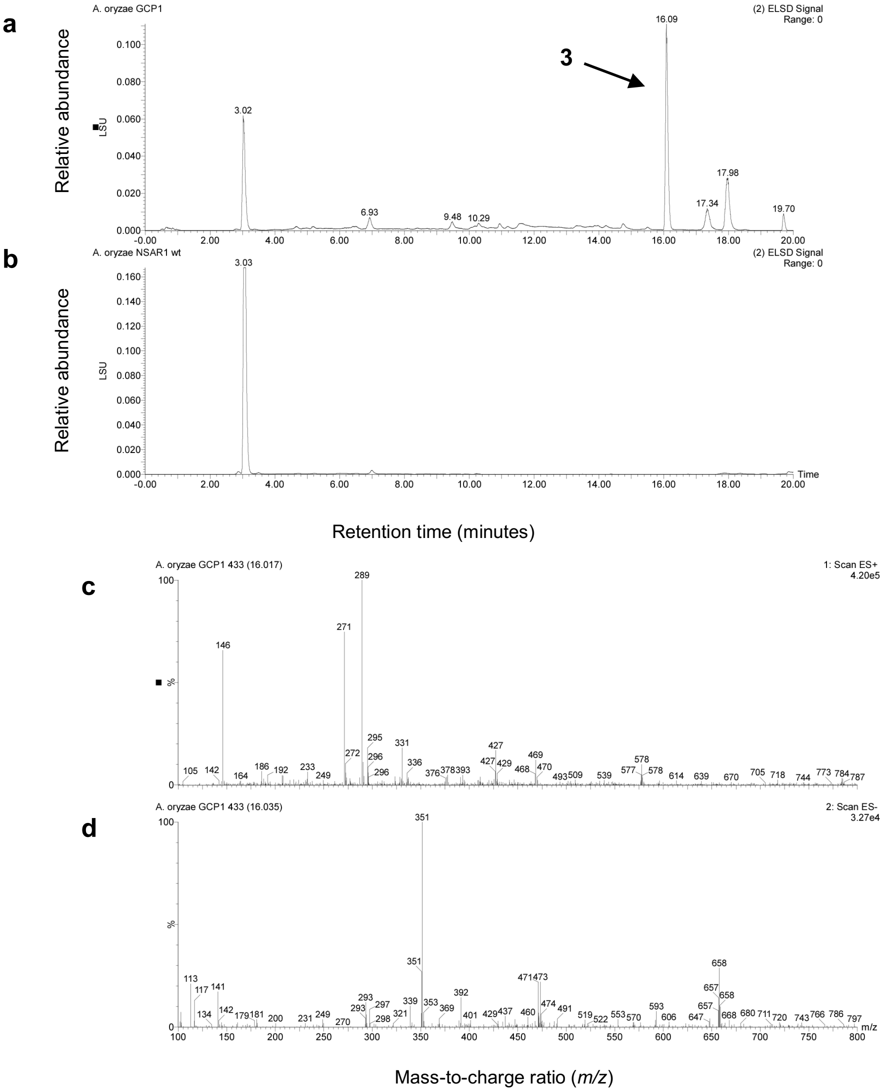

Supplementary Figure 11. HPLC-MS analysis of GCP1 extract. HPLC-MS traces of crude extracts from the transformant strain GCP1 (**a**) and the control strain *A. oryzae* NSAR1 (**b**). Traces report relative abundance of compounds, recorded through ELSD. Electrospray mass spectrometry of **3** in positive (**c**) and negative mode (**d**).

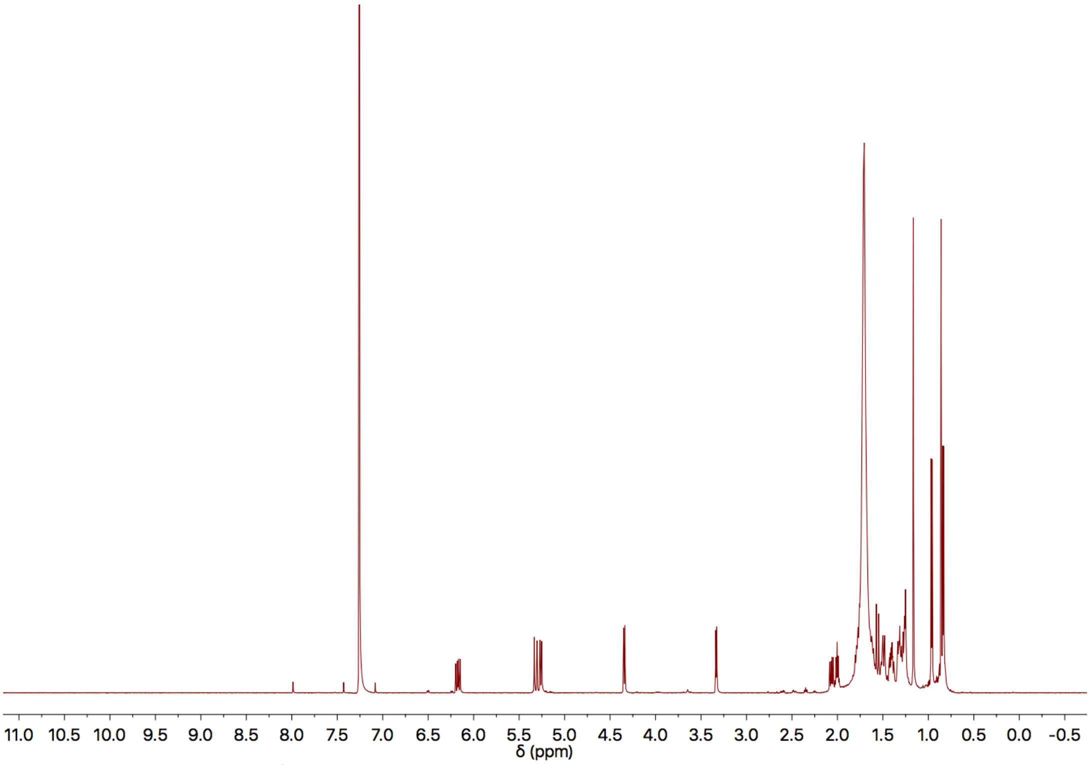

Supplementary Figure 12. ^{1}H-NMR spectrum of **3** in $CDCl_3$ (500 MHz).

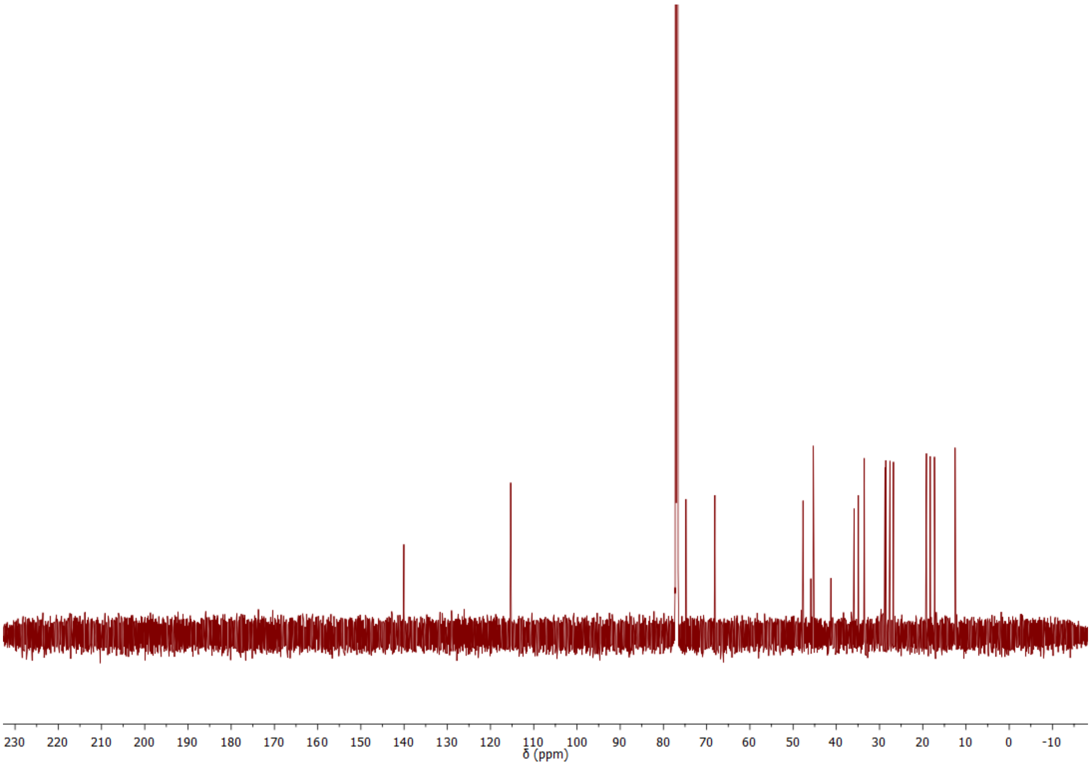

Supplementary Figure 13. ^{13}C-NMR spectrum of **3** in $CDCl_3$ (125 MHz).

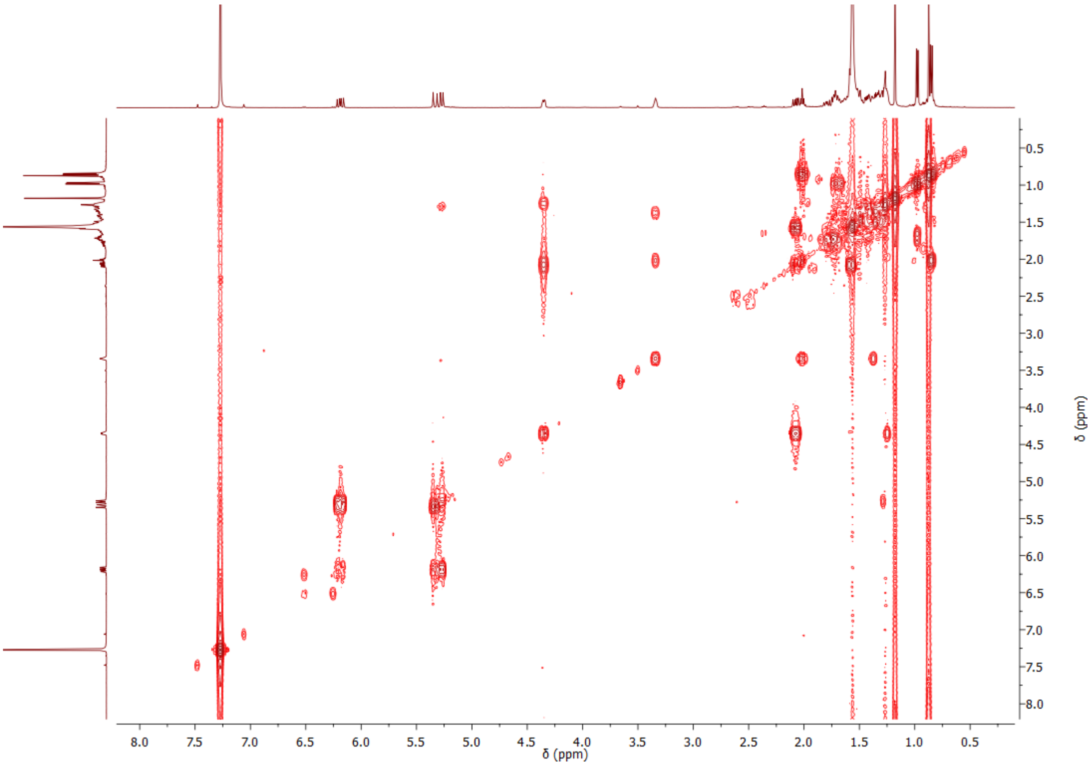

Supplementary Figure 14. COSY spectrum of **3** in $CDCl_3$ (500 MHz).

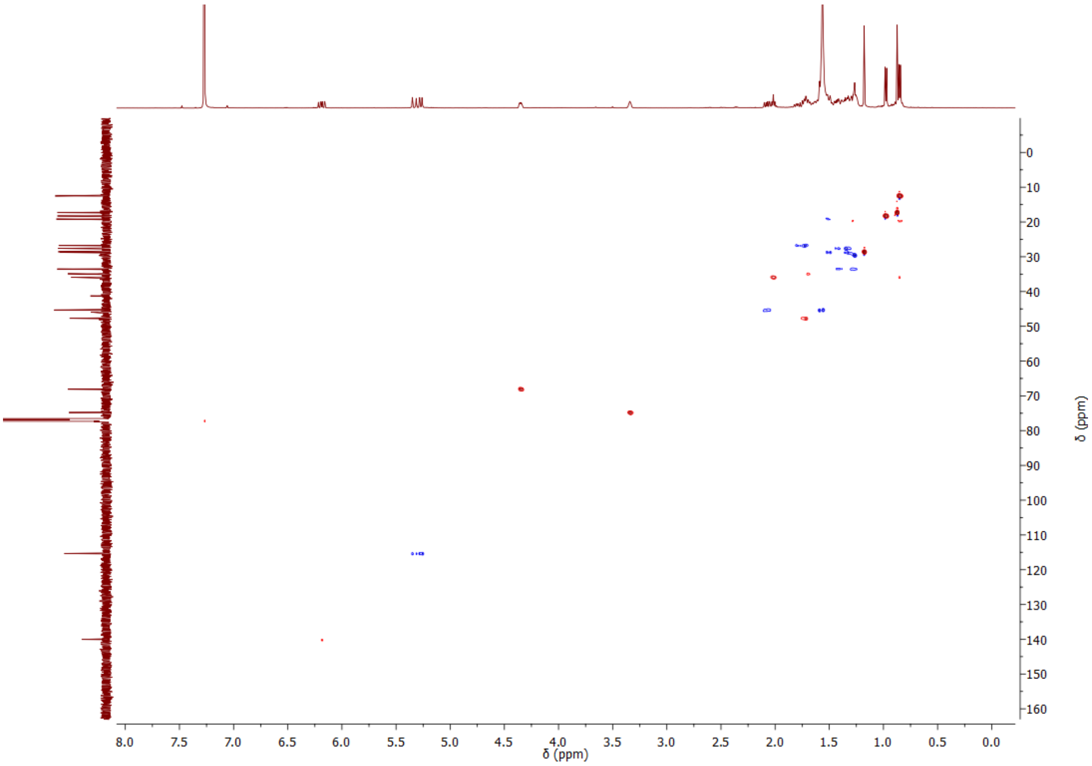

Supplementary Figure 15. HSQC spectrum of **3** in $CDCl_3$ (500 MHz).

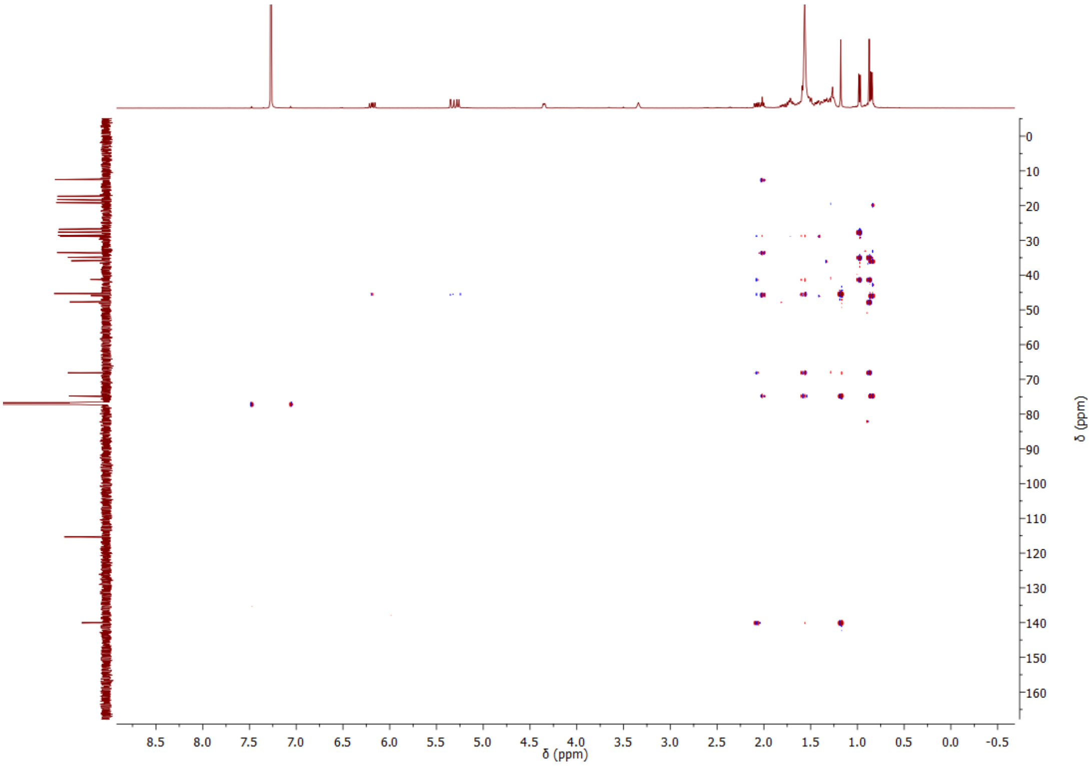

Supplementary Figure 16. HMBC spectrum of **3** in $CDCl_3$ (500 MHz).

Supplementary Table 2. NMR data assignment of **3** in $CDCl_3$. ESI-HRMS was used to determine the molecular formula of **3** (ESI-HRMS: *m/z* 329.2453 $[M+Na]^+$, calculated for $C_{20}H_{34}O_2Na^+$: 329.2451, Δ = 0.2 mmu).

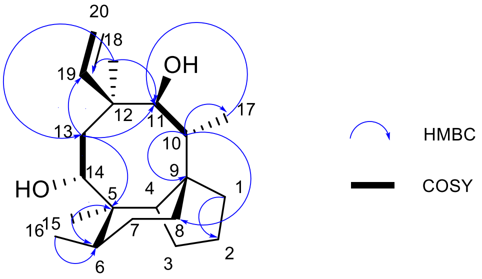

	Experimental data for **3**							Reference*
#	$^{13}C(\delta)$	HSQC	$^{1}H(\delta)$	*J* Coupling (Hz)	Protons	HMBC	COSY	$^{13}C(\delta)$
1	28.7	CH_2	1.24 – 1.30	m	1	C-2		28.8
			1.38 – 1.43	m	1	C-2		
2	19.2	CH_2	1.47 – 1.53	m	1			19.2
			1.59 – 1.65	m	1			
3	26.8	CH_2	1.60 – 1.64	m	1			26.8
			1.71 – 1.74	m	1			
4	47.7	CH	1.70 – 1.75	m	1			47.6
5	41.3			-				41.2
6	34.9	CH	1.68 – 1.72	m	2			34.9
7	27.6	CH_2	1.30 – 1.36	m	1			27.7
			1.40 – 1.44	m	1			
8	33.5	CH_2	1.30 – 1.36	m	1			33.5
			1.48 – 1.53	m	1			
9	45.9			-				45.9
10	35.9	CH	2.02	p, 6.9	1	C-8, C-9, C-17	H-11	36.0
11	74.8	CH	3.33	d, 6.4	1		H-10	74.5
12	45.3			-				45.5
13	45.4	CH_2	1.57 – 1.61	m	1	C-5, C-11, C-12, C-14, C-19		45.5
			2.08	dd, 15.6, 8.4	1	C-5, C-19	H-14	
14	68.1	CH	4.34	d, 8.3	1	C-14	H-13b	67.8
15	17.3	CH_3	0.87	s	3	C-4, C-5, C-6, C-14		17.4
16	18.3	CH_3	0.98	d, 7.1	3	C-5, C-6, C-7		18.3
17	12.5	CH_3	0.85	d, 7.1	3	C-9, C-11		12.6
18	28.6	CH_3	1.18	s	3	C-11, C-13, C-14, C-19		28.8
19	140.1	CH	6.17	dd, 17.8, 11.1	1		H-20a, H-20b	140.4
20	115.3	CH_2	5.26	dd, 11.1, 1.5	1		H-19	115.0
			5.32	dd, 17.8, 1.5	1			

*Reported ^{13}C-NMR chemical shifts for **3** from Schulz and Berner[2]

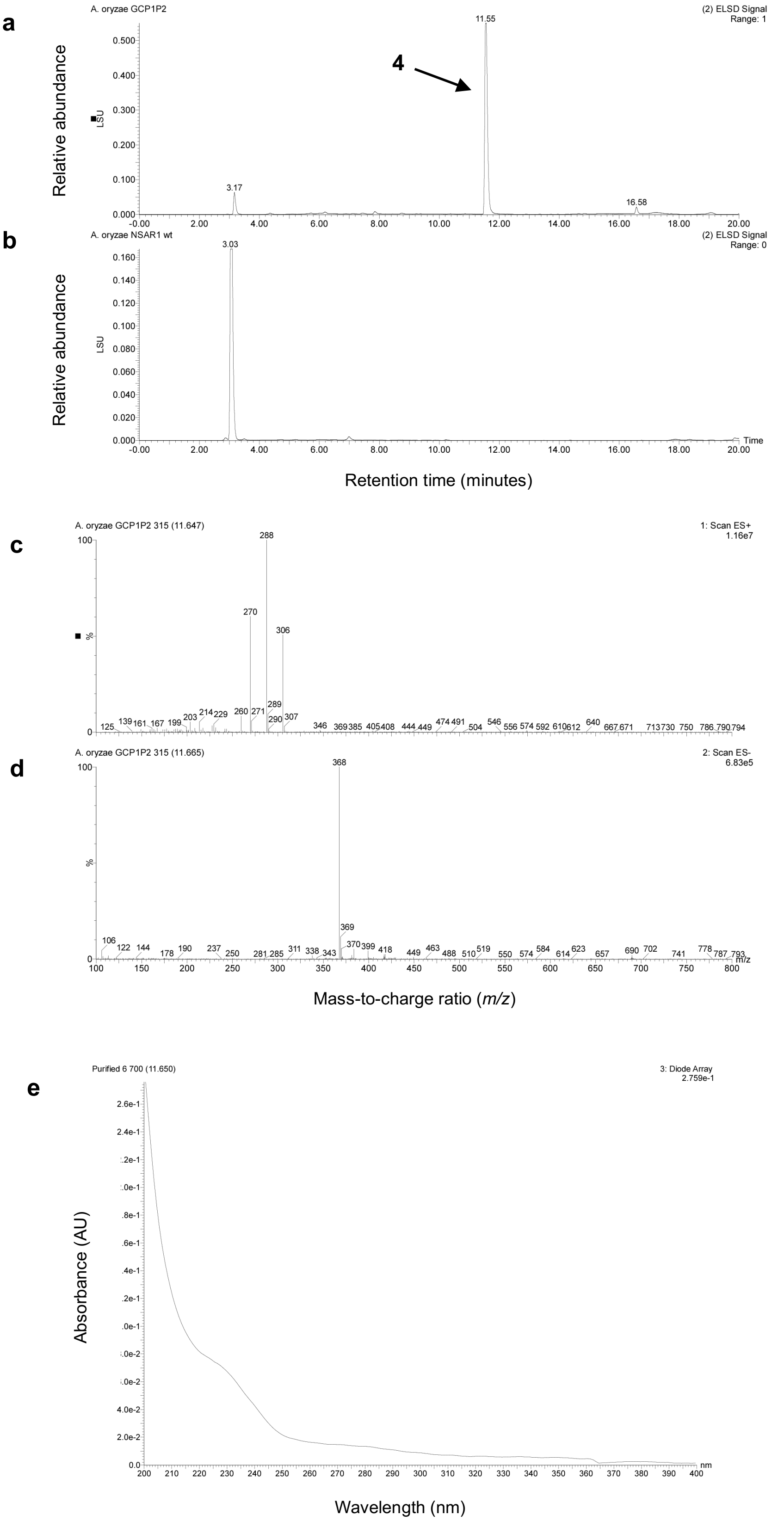

Supplementary Figure 17. HPLC-MS analysis of GCP1P2 extract. HPLC-MS traces of crude extracts from the *A. oryzae* transformant strain GCP1P2 (**a**) and the control strain *A. oryzae* NSAR1 (**b**). Traces report relative abundance of compounds, recorded through ELSD. Electrospray mass spectrometry of **4** in positive (**c**) and negative mode (**d**). UV absorption spectrum of **4** (**e**).

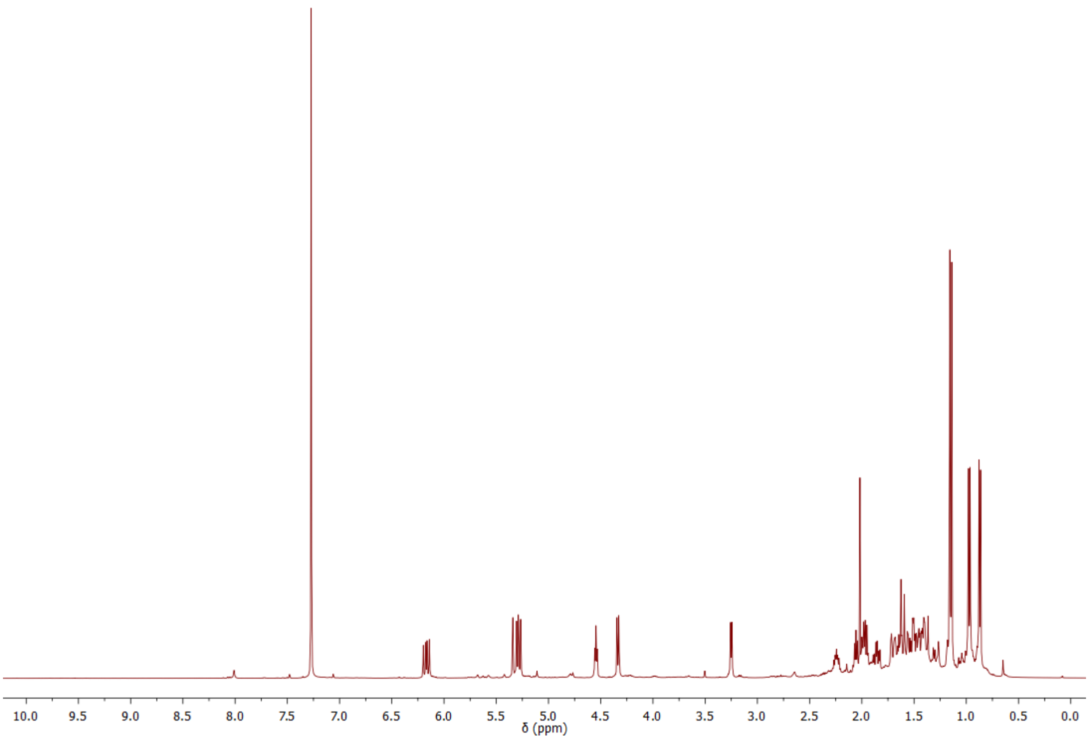

Supplementary Figure 18. ^{1}H-NMR spectrum of **4** in $CDCl_3$ (500 MHz)

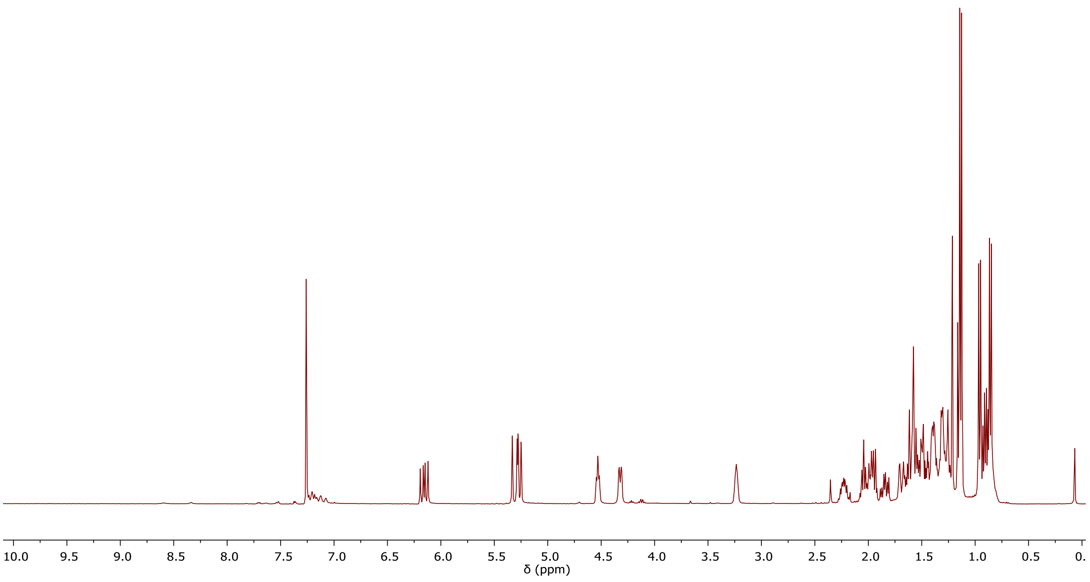

Supplementary Figure 19. ^{1}H-NMR spectrum of synthetic **4** in $CDCl_3$ (500 MHz).

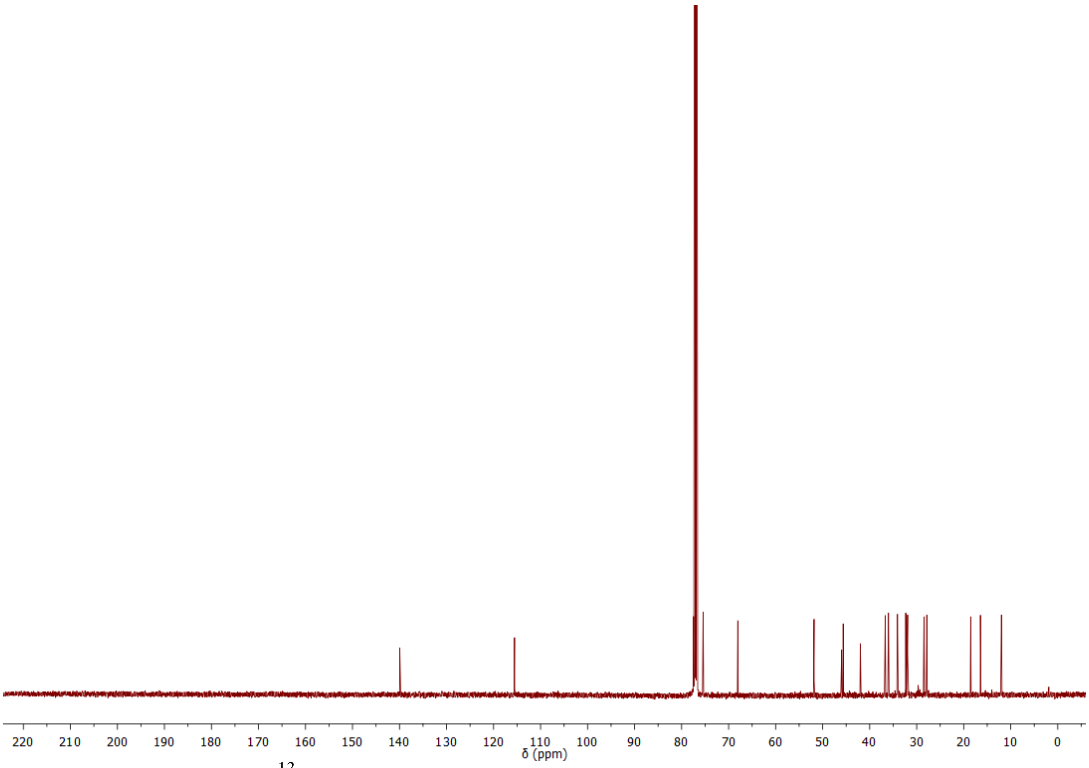

Supplementary Figure 20. ^{13}C-NMR spectrum of **4** in $CDCl_3$ (125 MHz).

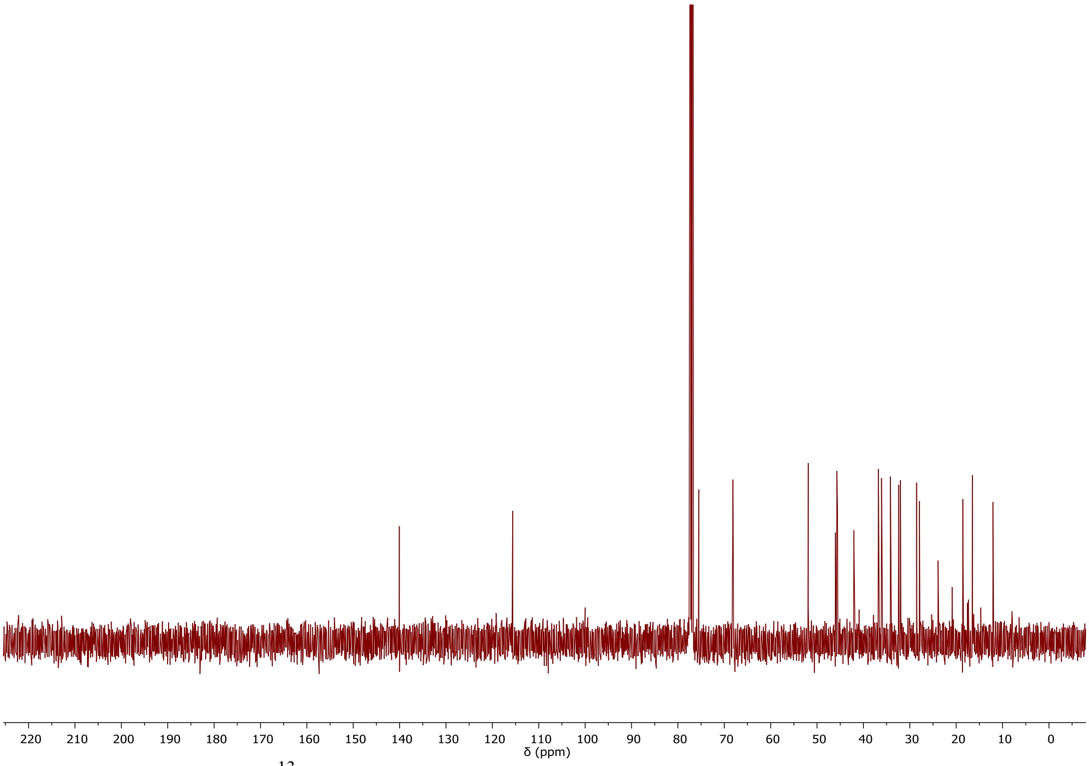

Supplementary Figure 21. ^{13}C-NMR spectrum of synthetic **4** in $CDCl_3$ (125 MHz).

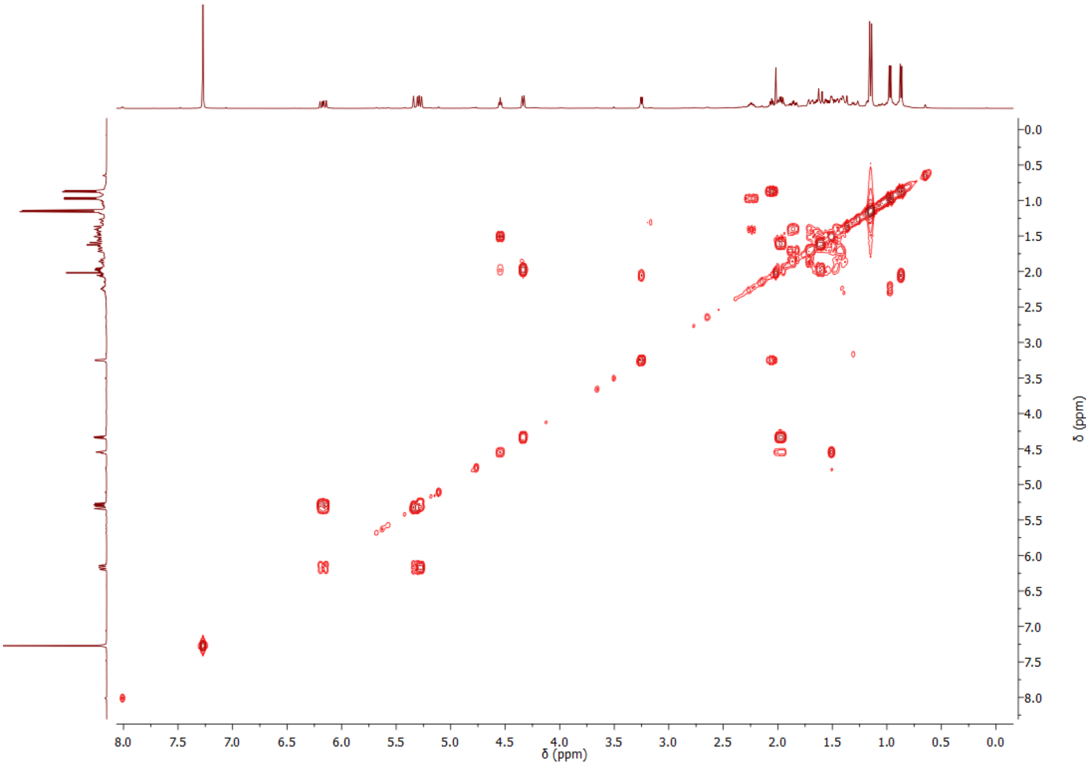

Supplementary Figure 22. COSY spectrum of **4** in $CDCl_3$ (500 MHz).

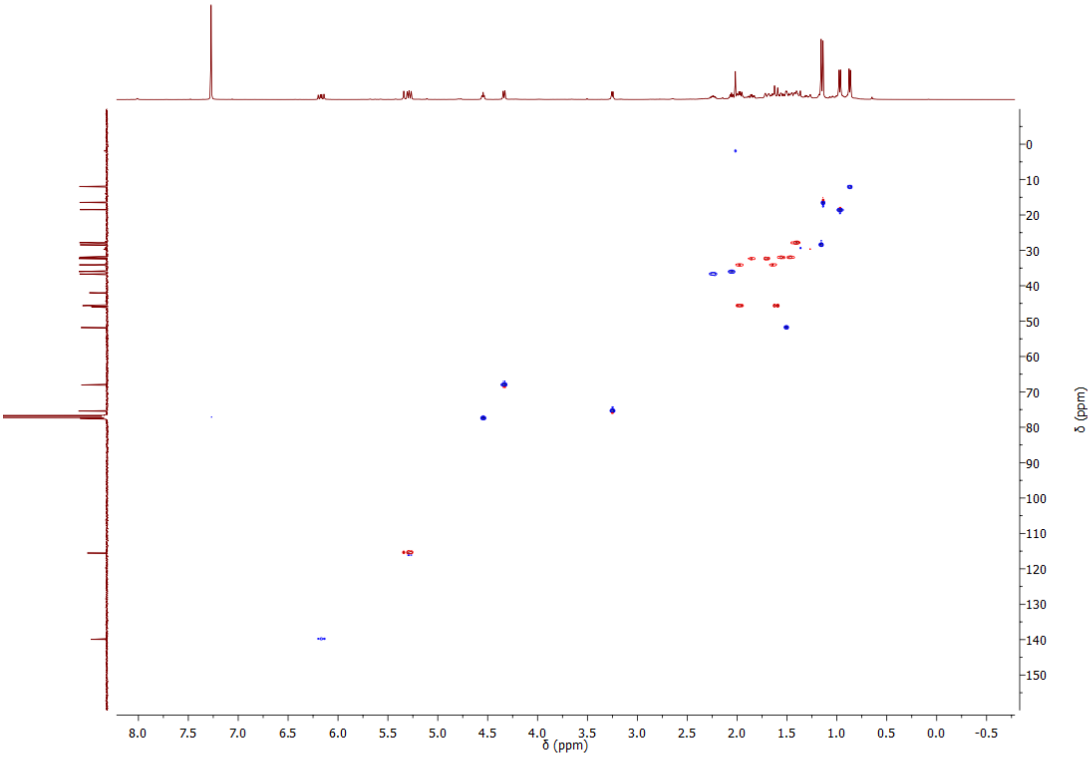

Supplementary Figure 23. HSQC spectrum of **4** in $CDCl_3$ (500 MHz).

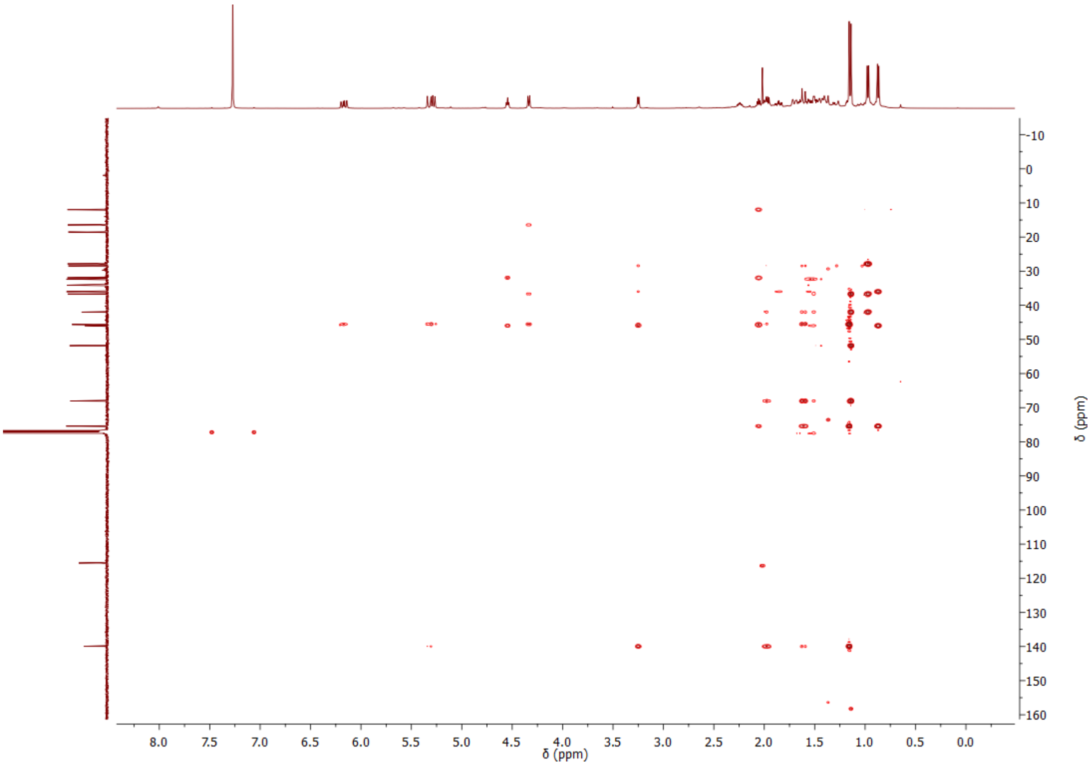

Supplementary Figure 24. HMBC spectrum of **4** in $CDCl_3$ (500 MHz).

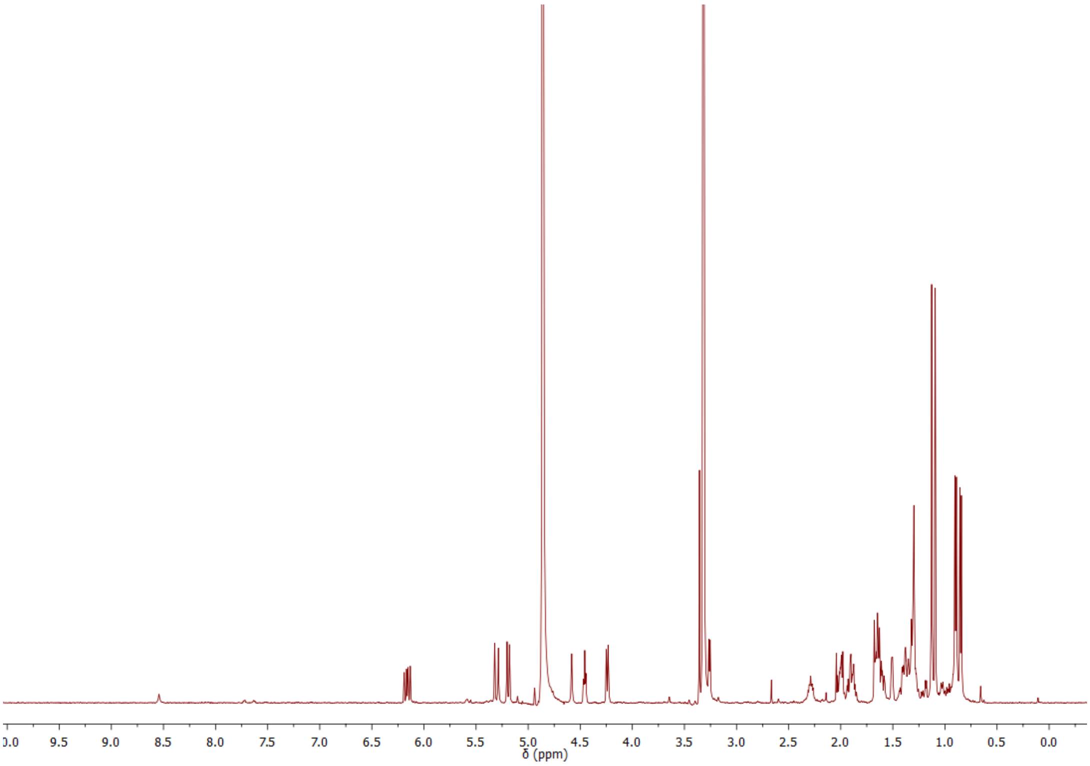

Supplementary Figure 25. ^{1}H-NMR spectrum of **4** in CD_3OD (500 MHz).

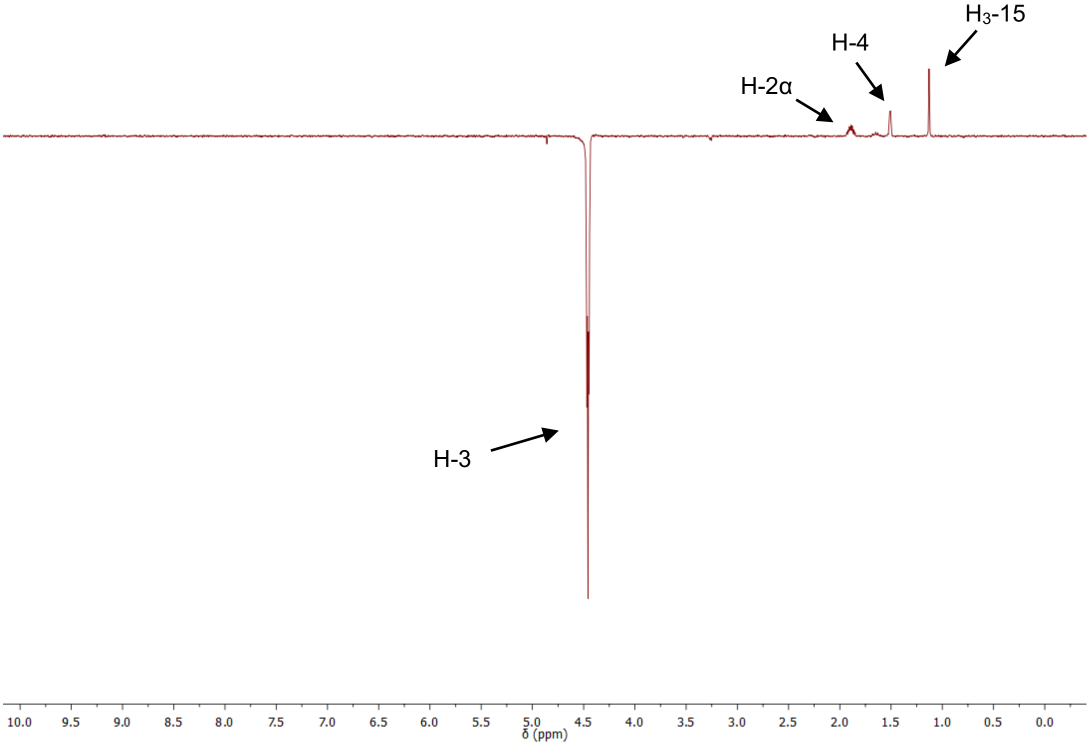

Supplementary Figure 26. Selective nOe of **4** in CD_3OD. The proton H-3 was selectively irradiated (4.45 ppm, corresponding to the signal 4.54 ppm for **4** in $CDCl_3$). The spectrum showed enhanced signals for protons H-2α, H-4 and H_3-15.

Supplementary Table 3. NMR data assignment of **4** in $CDCl_3$. ESI-HRMS was used to determine the molecular formula of **4** (ESI-HRMS: *m/z* 345.239499 $[M+Na]^+$, calculated for $C_{20}H_{34}O_3Na^+$: 345.240016, Δ = 0.517 mmu).

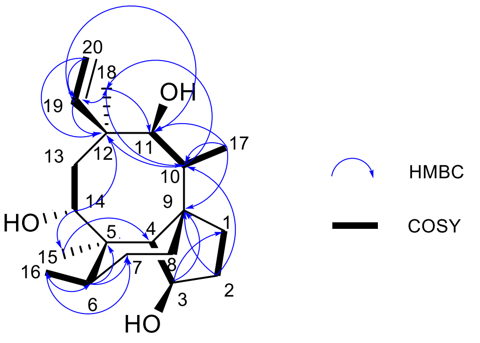

#	^{13}C(δ)	HSQC	^{1}H(δ)	*J* Coupling (Hz)	Protons	HMBC	COSY
1	32.3	CH_2	1.57-1.72	m	1		H-2b
			1.42	m	1		
2	32.7	CH_2	1.64-1.72	m	1		
			1.82-1.89	m	1	C-9, C-10	H-1a
3	77.9	CH	4.54	m	1	C-1, C-9	H-4
4	52.2	CH	1.50	dd, 5.4, 2.0	1		H-3
5	42.4						
6	37.1	CH	2.24	ddd, 11.7, 7.2, 4.8	1	C-5, C-7, C-14, C-16	H-16
7	28.3	CH_2	1.38-1.46	m	2		
8	34.5	CH_2	1.57-1.72	m	1		
			1.91-2.07	m	1		
9	46.4						
10	36.4	CH	2.06	p, 7.1	1	C-10, C-12, C-18	H-11, H-17
11	75.8	CH	3.25	d, 5.8	1		H-10
12	45.9						
13	46.0	CH_2	1.57-1.72	m	1		
			1.94-2.01	m	1		
14	68.5	CH	4.33	d, 8.6	1	C-6, C-12, C-15,	H-13b
15	16.7	CH_3	1.14	s	3	C-4, C-5, C-6, C-14	
16	18.9	CH_3	0.97	d, 7.2	3	C-5, C-6, C-7	H-6
17	12.4	CH_3	0.87	d, 7.1	3	C-9, C-10, C-11	H-10
18	28.9	CH_3	1.16	s	3	C-10, C-11, C-19	
19	140.4	CH	6.17	dd, 17.8, 11.1	1	C-11, C-12, C-18	H-20a, H-20b
20	116.0	CH_2	5.20	dd, 17.8, 1.5	1	C-12	H-19
			5.31	dd, 11.1, 1.5	1	C-12, C-19	H-19

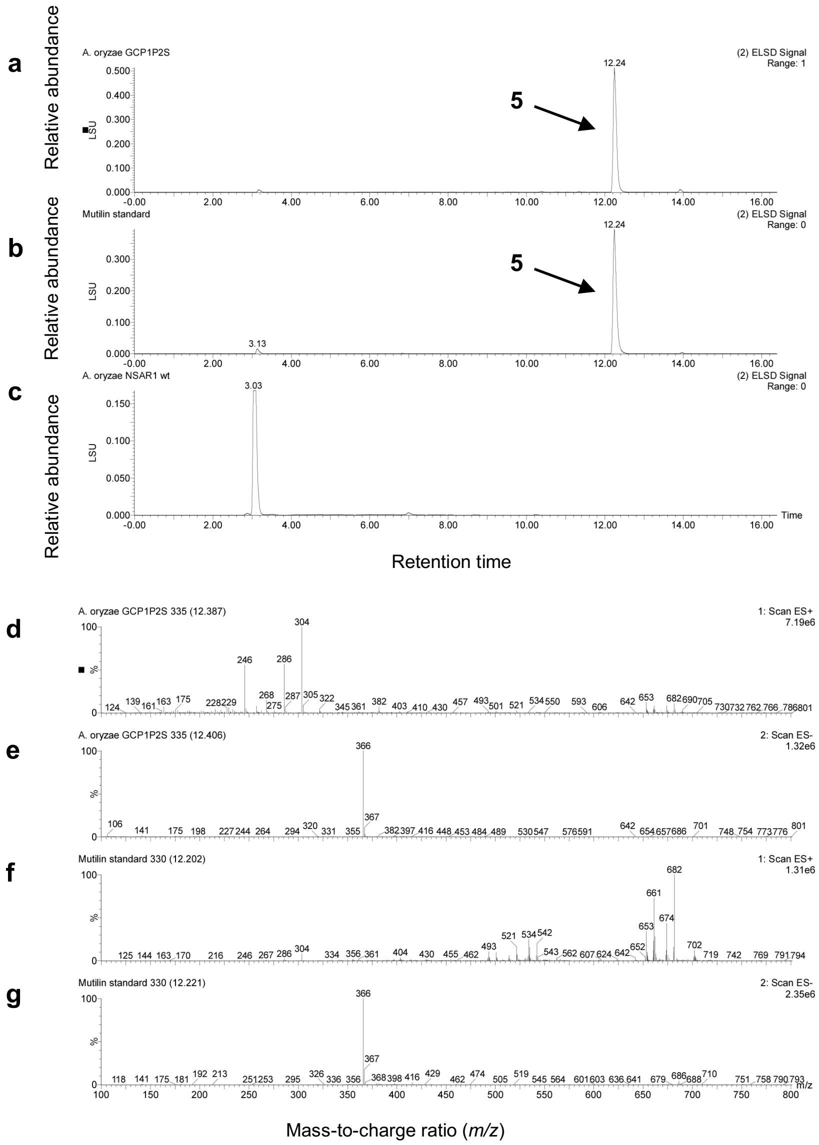

Supplementary Figure 27. HPLC-MS analysis of GCP1P2S extract. HPLC-MS traces of crude extracts from the *A. oryzae* transformant strain GCP1P2S (**a**), authentic **5** (**b**) and the control strain *A. oryzae* NSAR1 (**c**). Traces report relative abundance of compounds, recorded through ELSD. Electrospray mass spectrometry of **5** in *A. oryzae* strain GCP1P2S in positive (**d**) and negative mode (**e**), and of authentic **5** in positive (**f**) and negative mode (**g**).

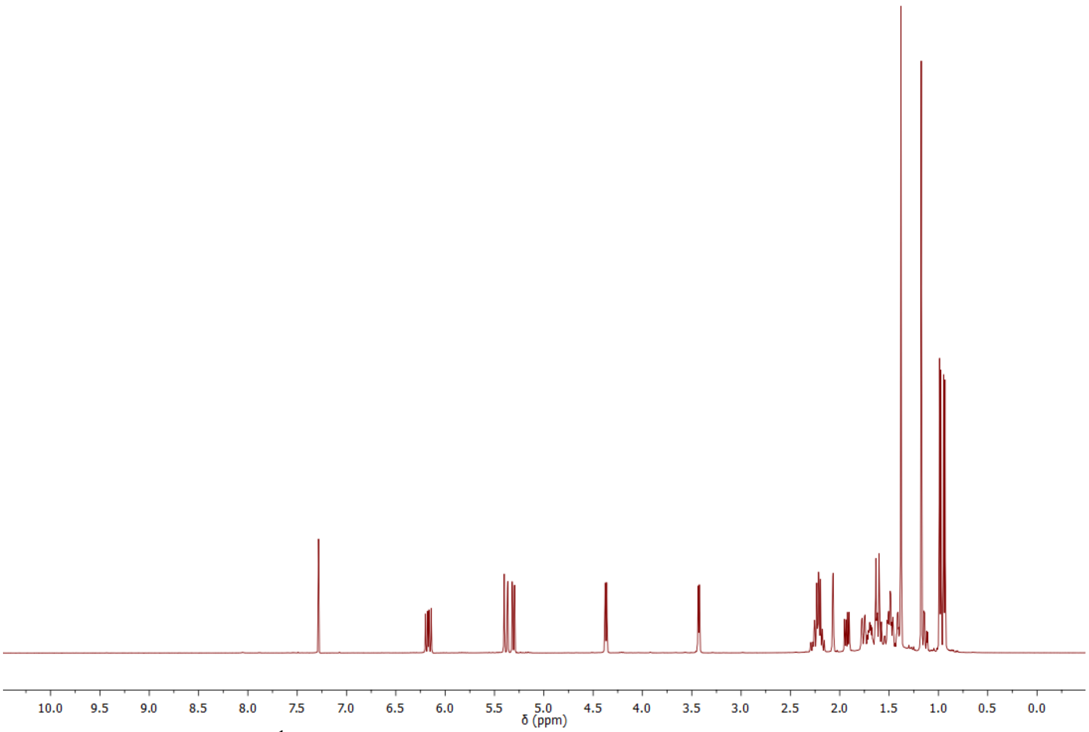

Supplementary Figure 28. ^{1}H-NMR spectrum of **5** in $CDCl_3$ (500 MHz).

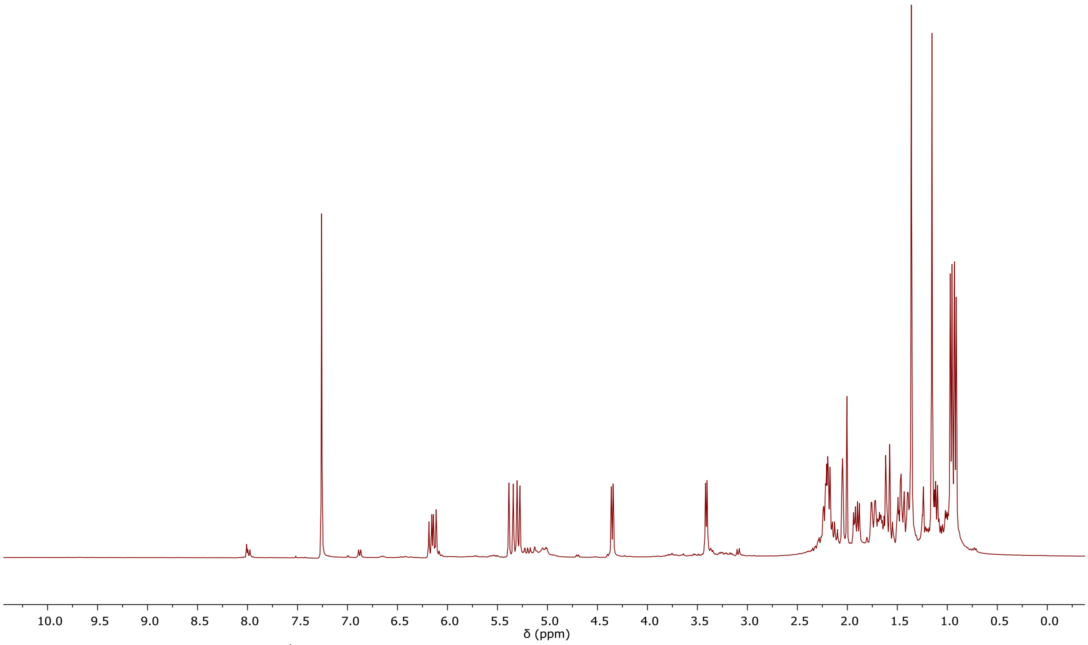

Supplementary Figure 29. ^{1}H-NMR spectrum of synthetic **5** in $CDCl_3$ (500 MHz).

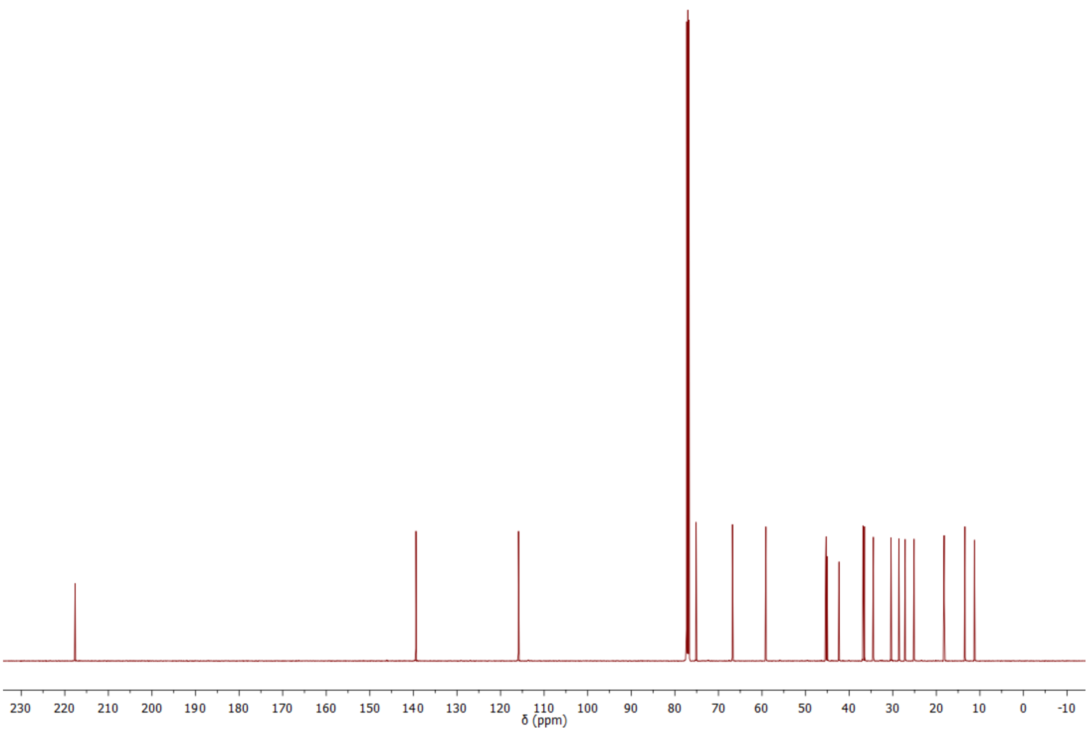

Supplementary Figure 30. ^{13}C-NMR spectrum of **5** in $CDCl_3$ (125 MHz).

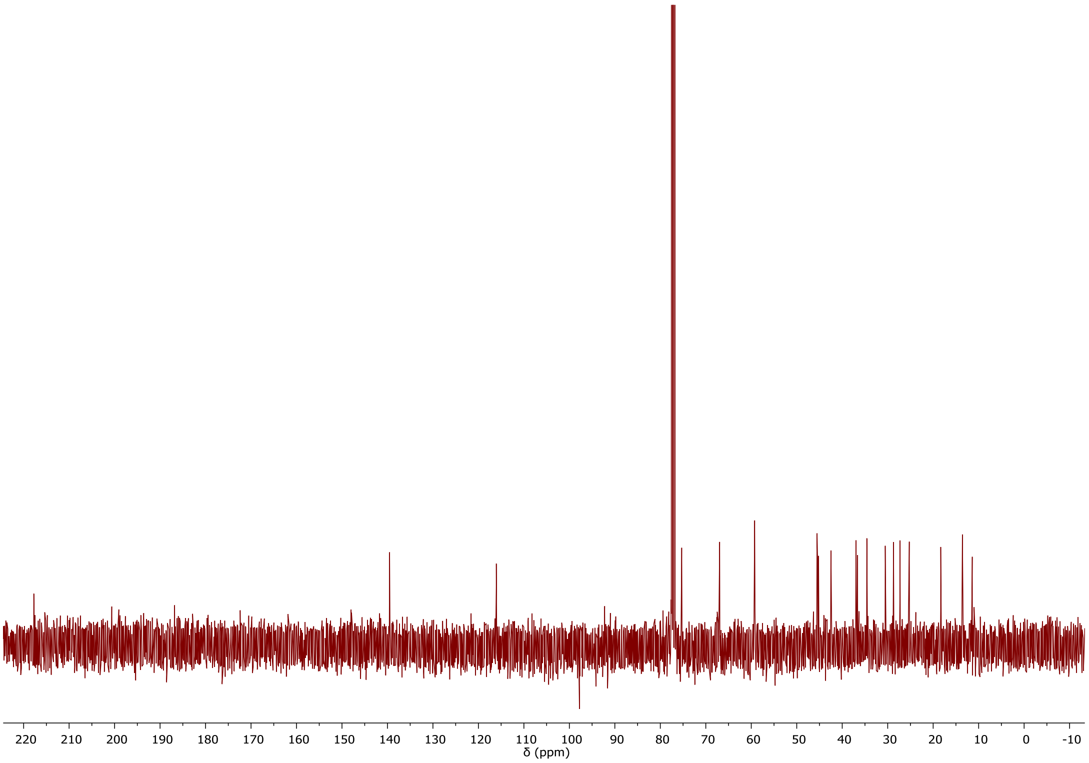

Supplementary Figure 31. ^{13}C-NMR spectrum of synthetic **5** in $CDCl_3$ (125 MHz).

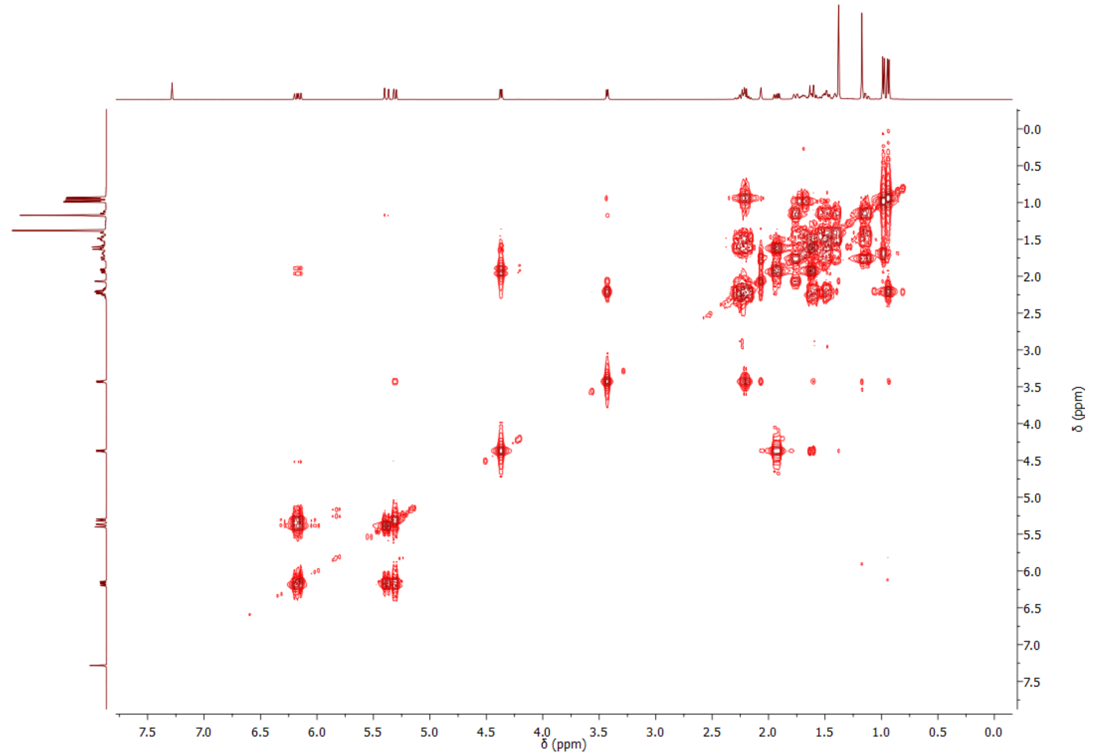

Supplementary Figure 32. COSY spectrum of **5** in $CDCl_3$ (500 MHz).

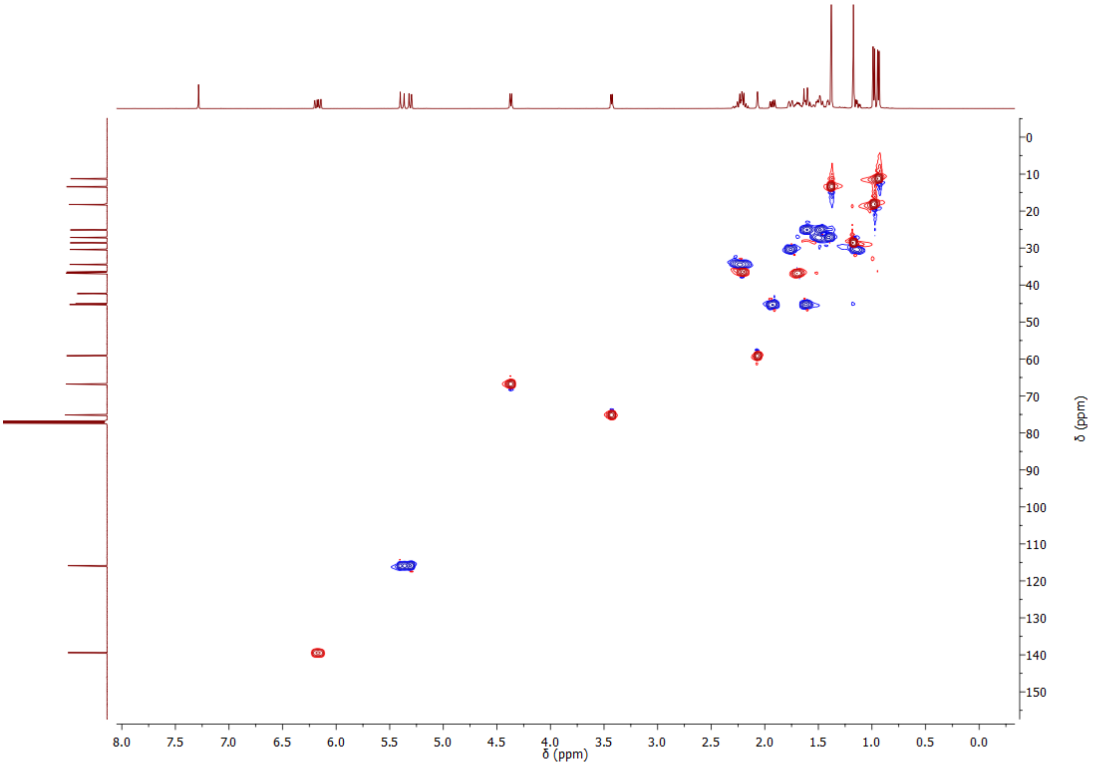

Supplementary Figure 33. HSQC spectrum of **5** in $CDCl_3$ (500 MHz).

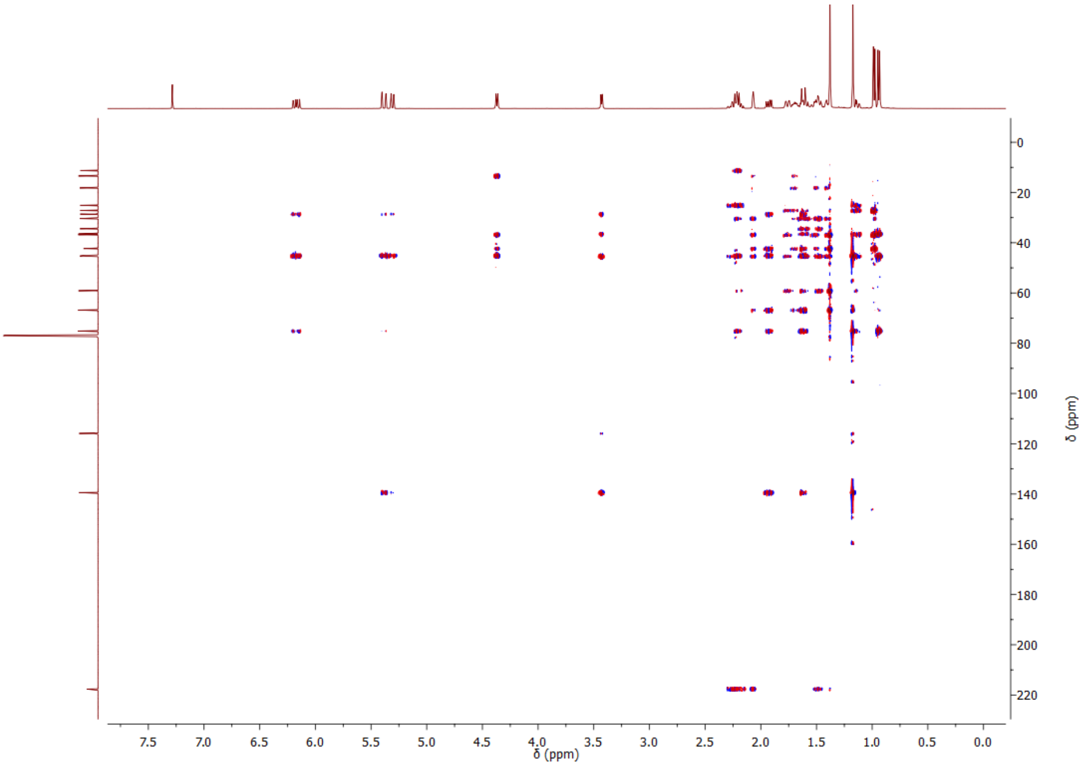

Supplementary Figure 34. HMBC spectrum of **5** in $CDCl_3$ (500 MHz).

Supplementary Table 4. NMR data assignment of **5** in $CDCl_3$. ESI-HRMS was used to determine the molecular formula of **7** (ESI-HRMS: *m/z* 343.2240 $[M+Na]^+$, calculated for $C_{20}H_{32}O_3Na^+$: 343.2244, Δ = 0.4 mmu).

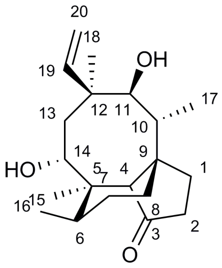

	Experimental data for **5**				Reference data for **5***			
#	^{13}C(δ)	^{1}H(δ)	*J* Coupling (Hz)	Protons	^{13}C(δ)	^{1}H(δ)	*J* Coupling (Hz)	Protons
1	25.0	1.45-1.52	m	1	25.1	1.39-1.79	m	2
		1.57-1.64	m	1				
2	34.3	2.13-2.29	m	2	34.4	2.14-2.31	m	2
3	217.5				217.6			
4	59.0	2.04	s	1	59.1	2.06	s	1
5	42.2				42.3			
6	36.7	1.62-1.70	m	1	36.8	1.39-1.79	m	1
7	27.0	1.36-1.44	m	1	27.1	1.39-1.79	m	2
		1.45-1.54	m	1				
8	30.3	1.84	dd, 14.5, 3.2	1	30.4	1.39-1.79	m	1
		1.11	dd, 14.0, 4.5	1		1.08-1.16	m	1
9	45.3				45.4			
10	36.4	2.13-2.29	m	1	36.4	2.14-2.31	m	1
11	75.0	3.41	d, 6.4	1	75.1	3.42	dd, 6.0, 5.9	1
12	45.0				45.0			
13	45.2	1.57-1.64	m	1	45.2	1.39-1.79	m	1
		1.90	dd, 15.8, 7.7	1		1.92	dd, 15.9, 7.7	1
14	66.7	4.35	d, 7.7	1	66.8	4.36	dd, 7.3, 5.7	1
15	13.3	1.36	s	3	13.4	1.37	s	3
16	18.1	0.96	d, 7.1	3	18.2	0.97	d, 7.3	3
17	11.1	0.92	d, 7.1	3	11.3	0.93	d, 7.3	3
18	28.5	1.15	s	3	28.5	1.16	s	3
19	139.3	6.15	dd, 17.7, 11.2	1	139.3	6.16	dd, 17.8, 11.1	1
20	115.8	5.28	dd, 11.2, 1.4	1	115.9	5.30	dd, 11.1, 1.4	1
		5.36	dd, 17.7,1.4	1		5.38	dd, 17.8, 1.4	1

*Reported ^{1}H-NMR ^{13}C-NMR chemical shifts for **5** from Fazakerley, *et al.*[3]

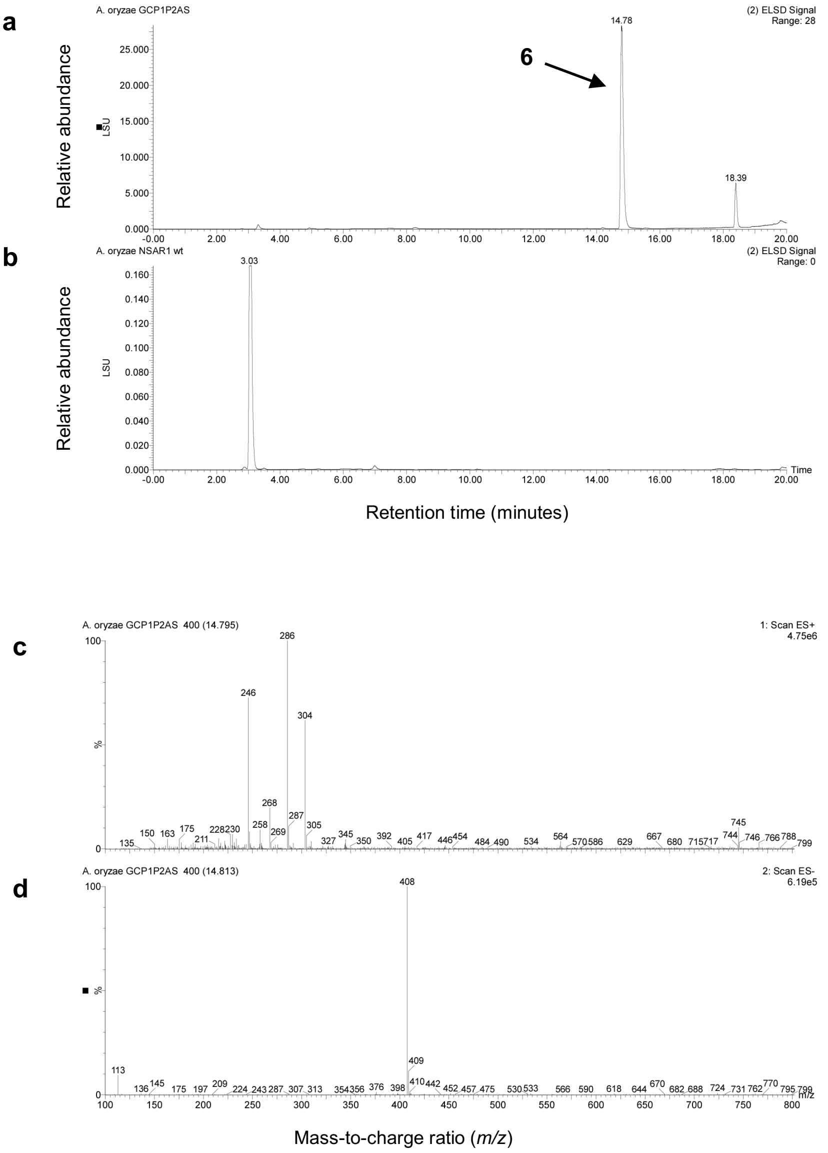

Supplementary Figure 35. HPLC-MS analysis of GCP1P2SA extract. HPLC-MS traces of crude extracts from the transformant strain GCP1P2SA (**a**), and control strain *A. oryzae* NSAR1 (**b**). Traces report relative abundance of compounds, recorded through ELSD. Electrospray mass spectrometry of **6** in positive (**c**) and negative mode (**d**).

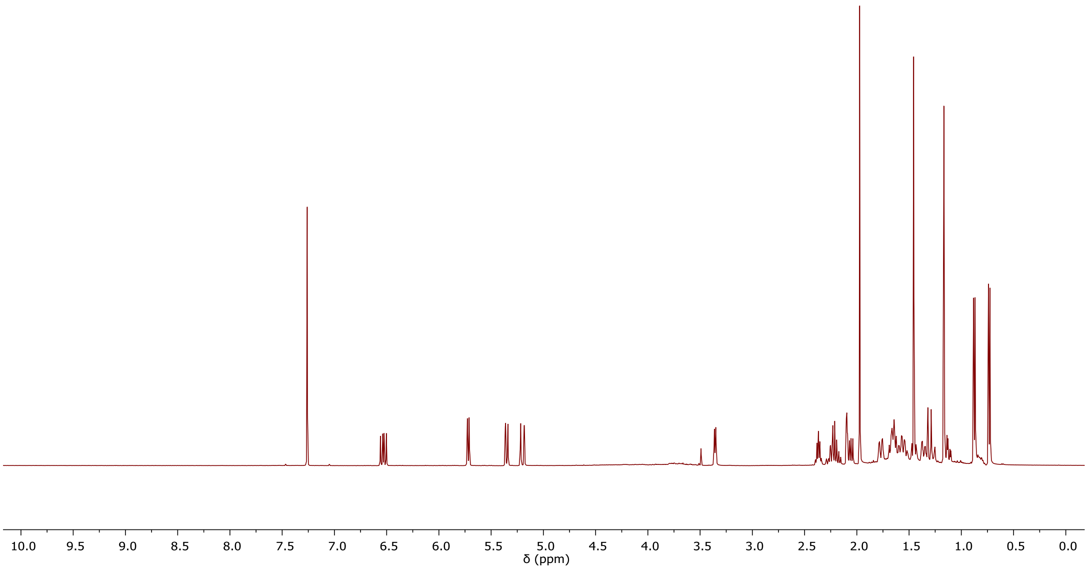

Supplementary Figure 36. ^{1}H-NMR spectrum of **6** in $CDCl_3$ (500 MHz).

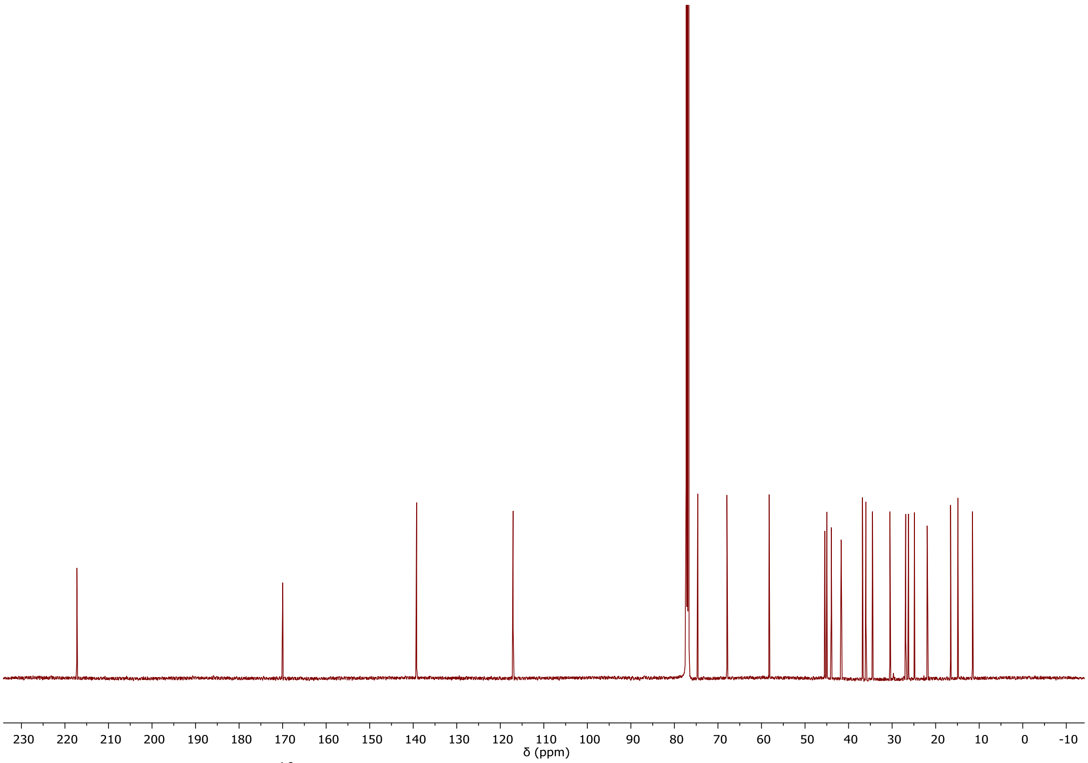

Supplementary Figure 37. ^{13}C-NMR spectrum of **6** in $CDCl_3$ (125 MHz).

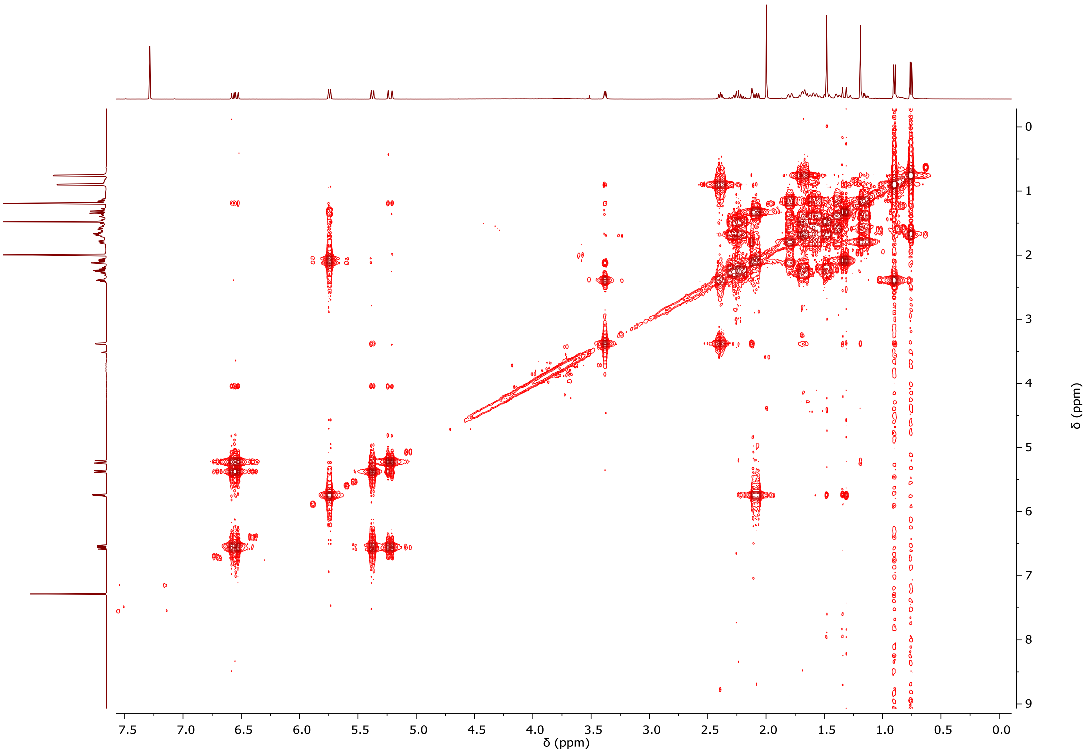

Supplementary Figure 38. COSY spectrum of **6** in $CDCl_3$ (500 MHz).

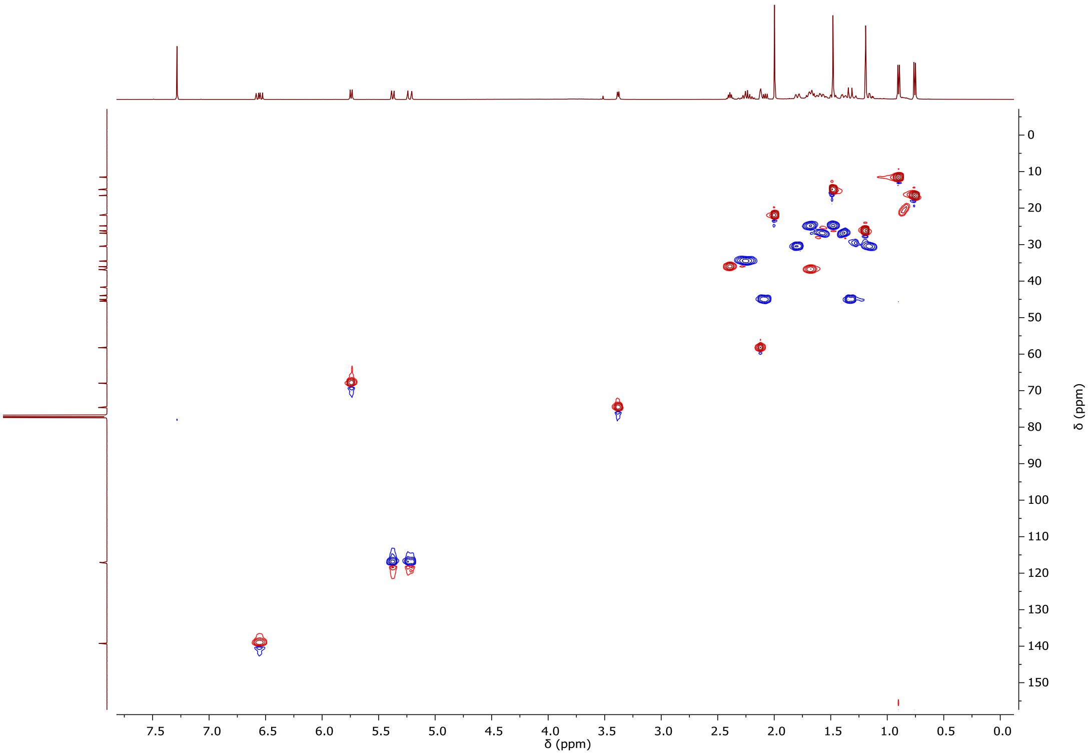

Supplementary Figure 39. HSQC spectrum of **6** in $CDCl_3$ (500 MHz).

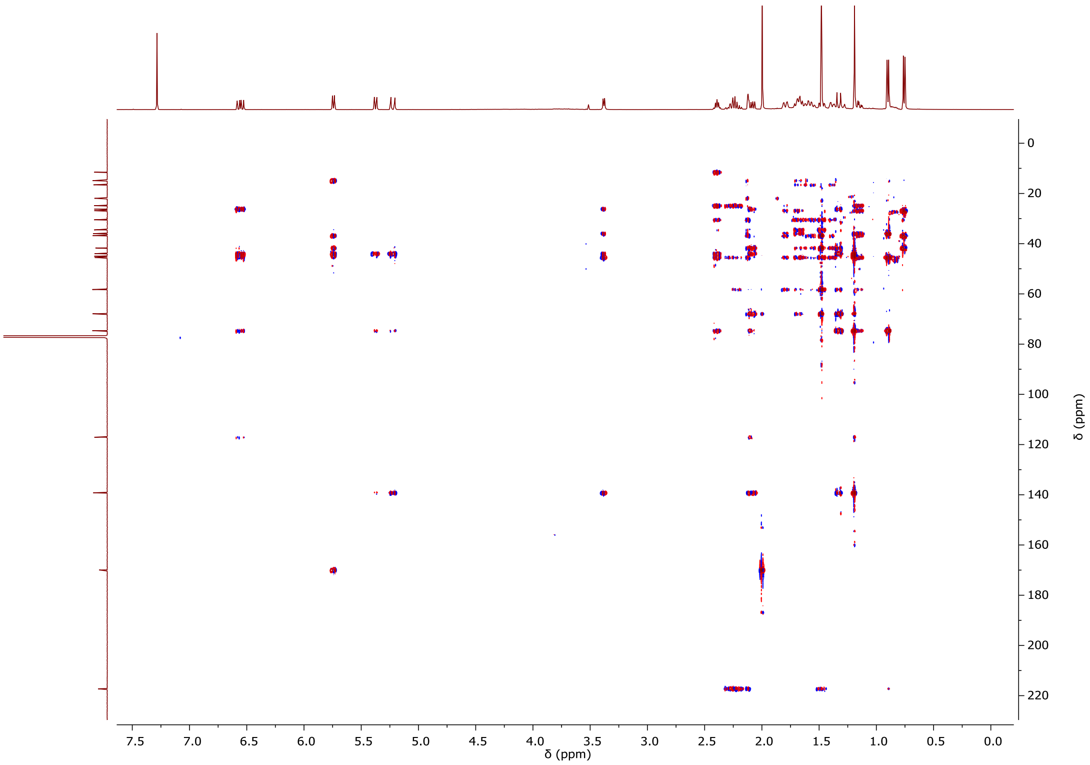

Supplementary Figure 40. HMBC spectrum of **6** in $CDCl_3$ (500 MHz).

Supplementary Table 5. NMR data of **6** in $CDCl_3$. ESI-HRMS was used to determine the molecular formula of **6** (ESI-HRMS: *m/z* 385.2346 $[M+Na]^+$, calculated for $C_{22}H_{34}O_4Na^+$: 385.2349, Δ = 0.3 mmu).

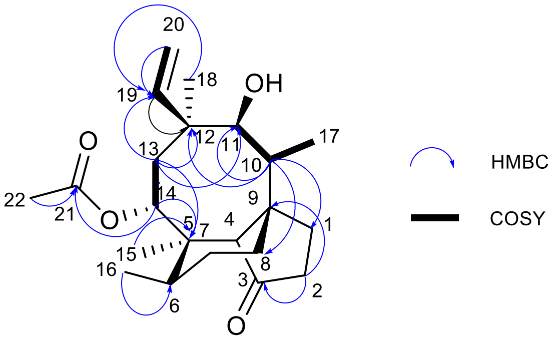

#	$^{13}C(\delta)$	HSQC	$^{1}H(\delta)$	*J* Coupling (Hz)	Protons	HMBC	COSY
1	24.9	CH_2	1.45	m	1		
			1.64	m	1	C-2, C-8	
2	34.5	CH_2	2.23	m	2	C-1, C-3, C-4, C-9	
3	217.2						
4	58.2	CH	2.07	m	1	C-3, C-5, C-8, C-14	
5	41.7						
6	36.8	CH	1.64	m	1	C-7, C-8	
7	26.9	CH_2	1.34	m	1		
			1.53	m	1		
8	30.5	CH_2	1.12	m	1		
			1.77	m	1	C-22	
9	45.5						
10	36.0	CH	2.37	p, 7.0	1		H-17
11	74.6	CH	3.36	m	1	C-1, C-8, C-11, C-12, C-13, C-17	H-10
12	44.0						
13	45.0	CH_2	1.30	d, 16.0	1	C-5, C-11, C-12, C-14, C-18, C-19	
			2.07	m	1	C-5, C-18, C-19	H-14
14	67.9	CH	5.72	d, 8.5	1	C-5, C-6, C-13, C-15	H-20a
15	14.9	CH_3	1.45	s	3	C-3, C-4, C-5, C-6, C-14,	
16	16.6	CH_3	0.73	d, 7.3	3	C-5, C-6, C-7	
17	11.5	CH_3	0.87	d, 7.0	3	C-9, C-10, C-11	H-10
18	26.2	CH_3	1.17	s	3	C-11, C-13, C-19	
19	139.2	CH	6.53	dd, 17.4, 11.0	1	C-11, C-12, C-18	H20a, H-20b
20	117.0	CH_2	5.20	dd, 17.4, 1.6	1	C-12, C-19	H-19
			5.35	dd, 11.0, 1.6	1	C-12	H-19
21	169.6						
22	21.9	CH_3	1.97	s	3	C-21	

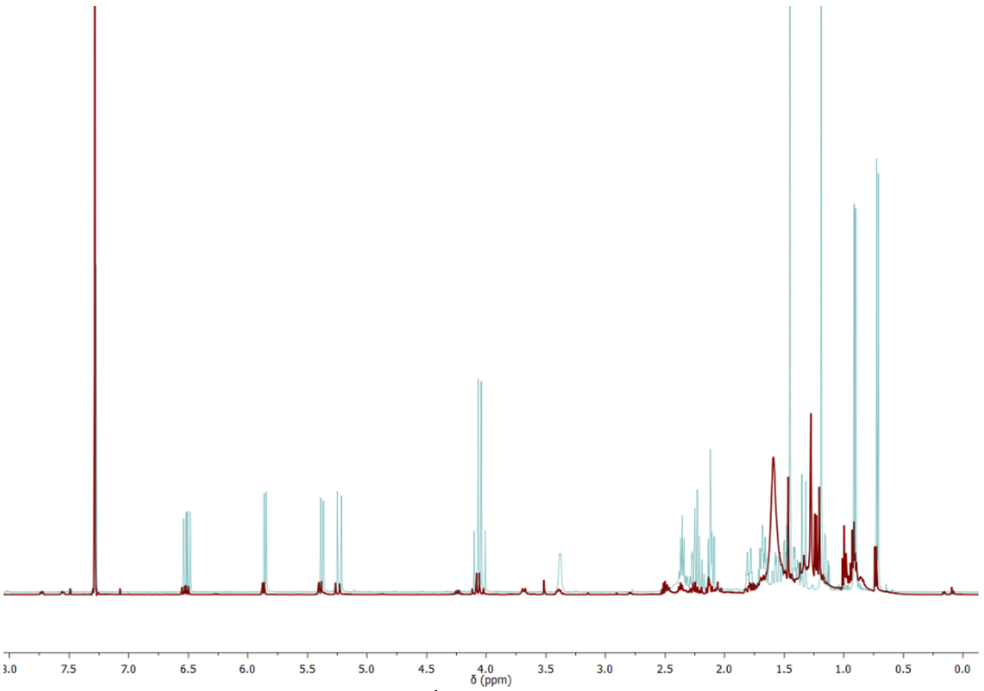

Supplementary Figure 41. Superimposed ^{1}H-NMR (500 MHz) spectra for **1** isolated from AP3 fed with **5** (in red) and authentic **1** (in light blue) in $CDCl_3$, showing conversion of **5** to **1** upon activity of Pl-atf and Pl-p450-3.

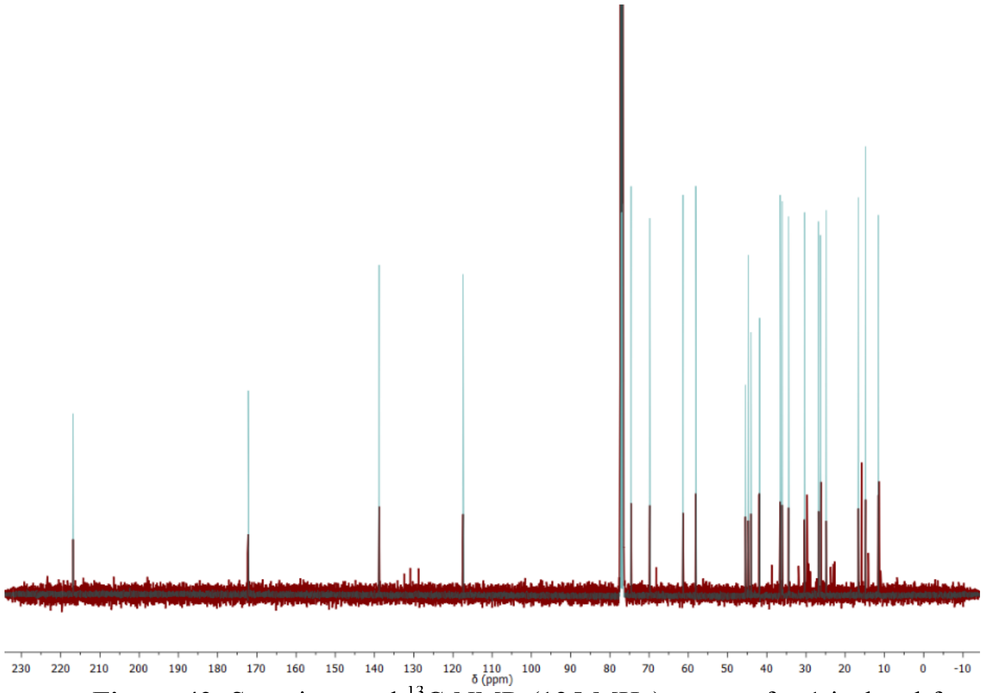

Supplementary Figure 42. Superimposed ^{13}C-NMR (125 MHz) spectra for **1** isolated from AP3 fed with **5** (in red), and authentic **1** (in light blue) in $CDCl_3$, showing conversion of **5** to **1** upon activity of Pl-atf and Pl-p450-3.

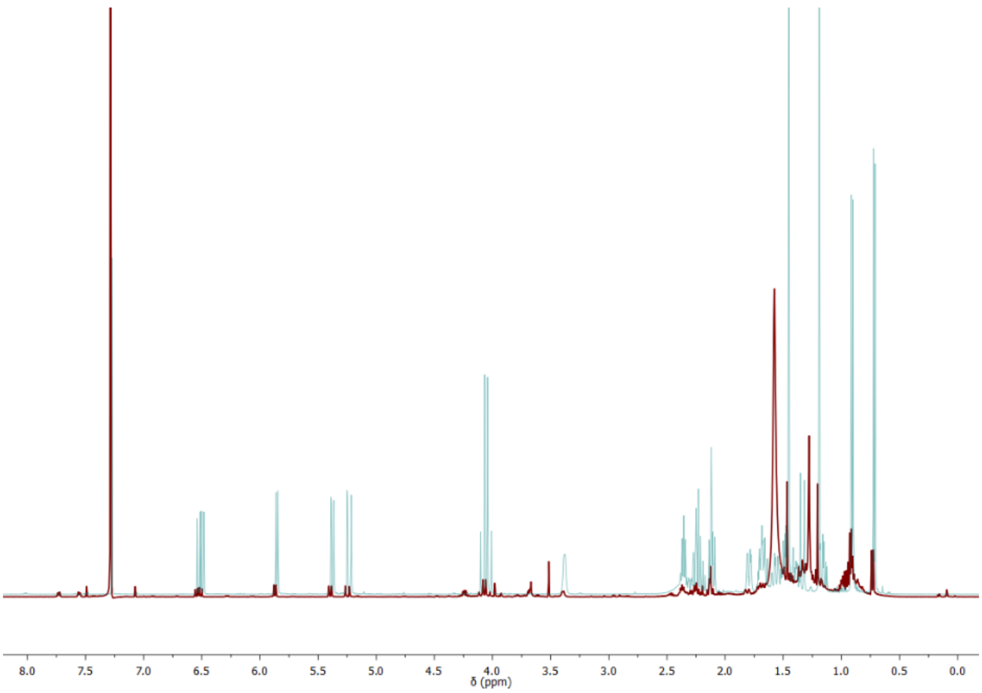

Supplementary Figure 43. Superimposed ^{1}H-NMR (500 MHz) spectra for **1** isolated from SAP3 fed with **4** (in red), and authentic **1** (in light blue) in $CDCl_3$, showing conversion of **4** to **1** upon activity of Pl-sdr, Pl-atf and Pl-p450-3.

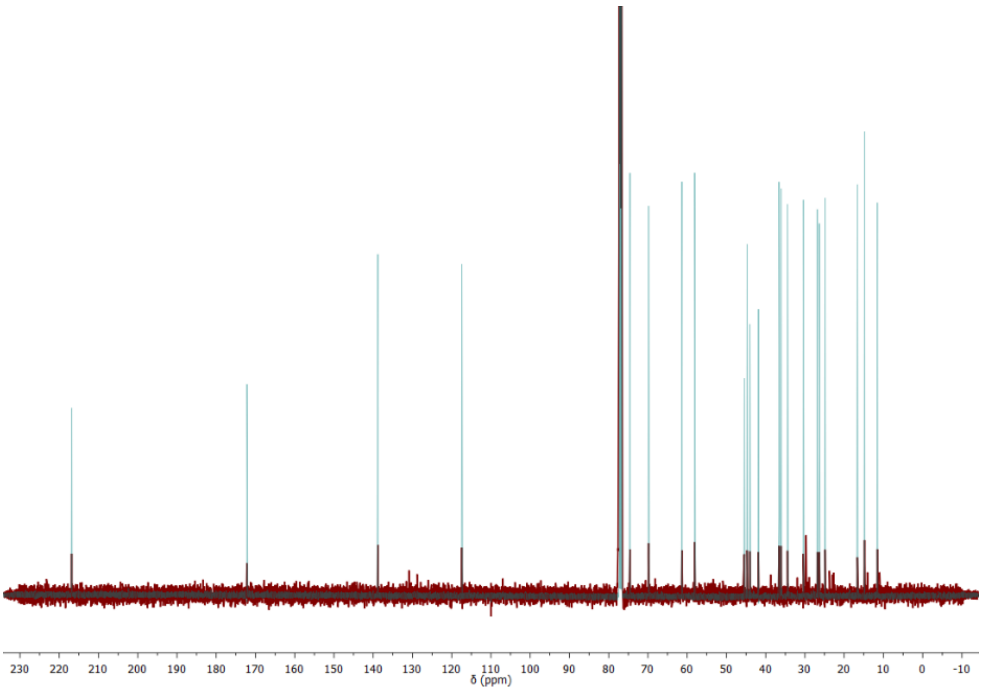

Supplementary Figure 44. Superimposed ^{13}C-NMR (125 MHz) spectra for **1** isolated from SAP3 fed with **4** (in red), and authentic **1** (in light blue) in $CDCl_3$, showing conversion of **4** to **1** upon activity of Pl-sdr, Pl-atf and Pl-p450-3.

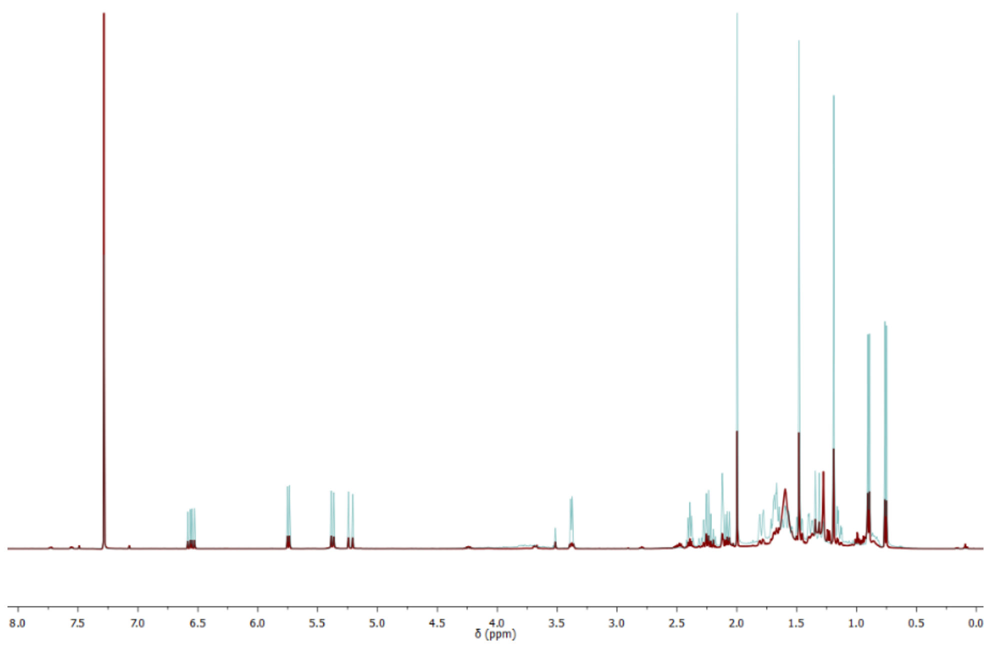

Supplementary Figure 45. Superimposed ^{1}H-NMR (500 MHz) spectra for **6** isolated from SAP3 fed with **4** (in red), and authentic **6** (in light blue) in $CDCl_3$, showing conversion of **4** to **6** upon activity of Pl-sdr, Pl-atf and Pl-p450-3.

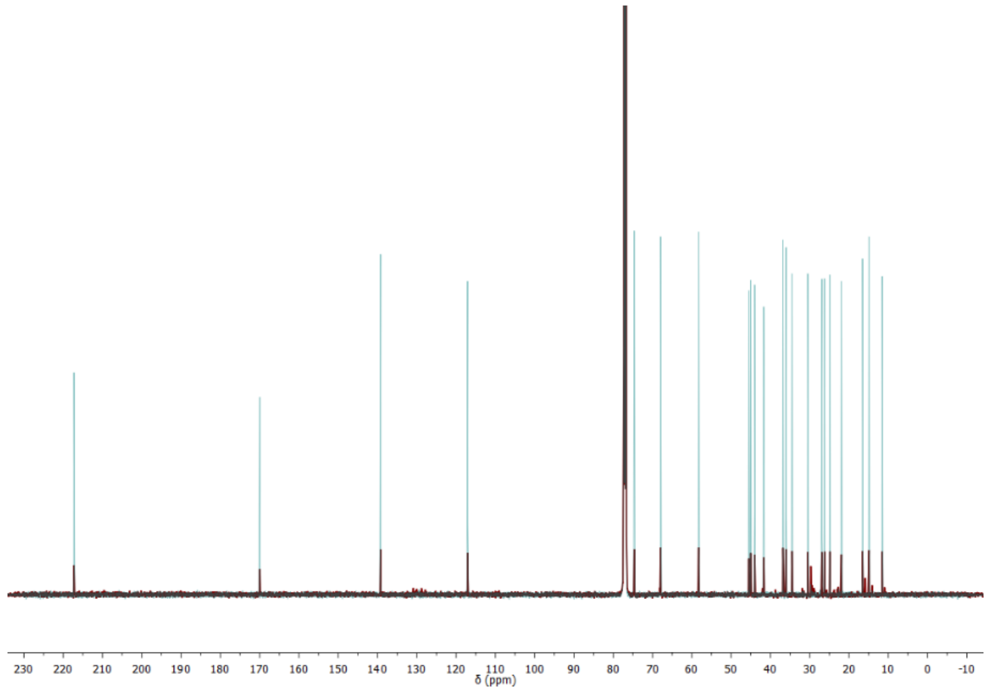

Supplementary Figure 46. Superimposed ^{13}C-NMR (125 MHz) spectra for **6** isolated from SAP3 fed with **4** (in red), and authentic **6** (in light blue) in $CDCl_3$, showing conversion of **4** to **6** upon activity of Pl-sdr, Pl-atf and Pl-p450-3.

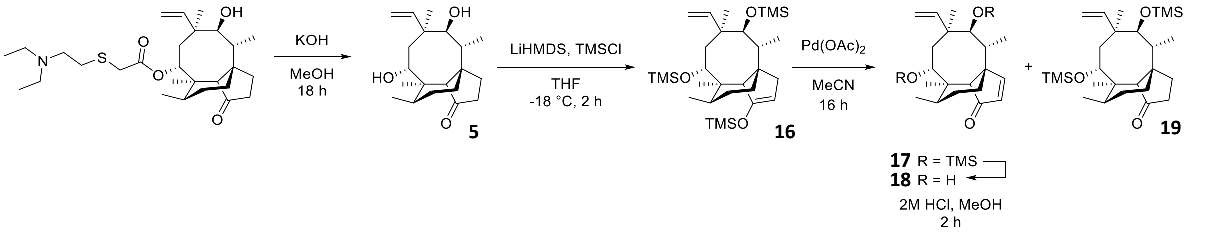

Supplementary Figure 47. Scheme representing synthetic conversion of tiamulin into enone **18**. First, the C14 side-chain was removed though hydrolysis using potassium hydroxide, to yield mutilin **5**. Formation of silyl enol ether **16** was then achieved using the procedure developed by Wang, *et al* [4]. **16** was then treated with palladium(II) acetate to generate the enone *via* a Saegusa–Ito oxidation. The TMS groups were then deprotected using 2M HCl.

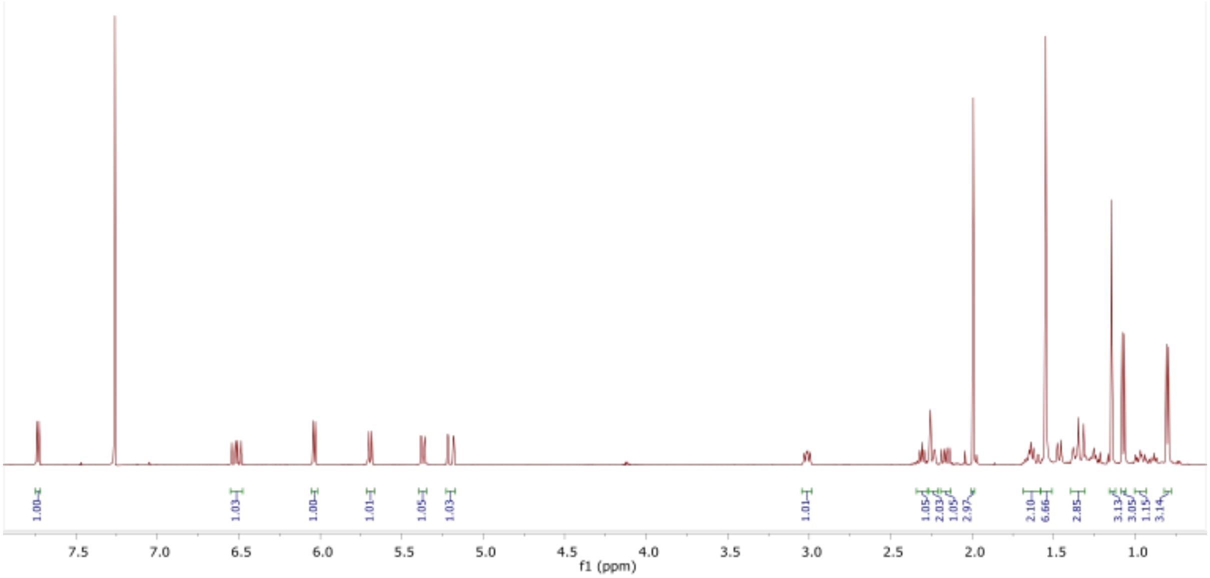

Supplementary Figure 48. ^{1}H-NMR spectrum of **20** in $CDCl_3$ (500 MHz). δ_H (500 MHz, $CDCl_3$) 7.73 (1H, d, *J* 6.0, 1-H), 6.51 (1H, dd, *J* 17.0, 11.0, 19-H), 6.04 (1H, d, *J* 6.0, 2-H), 5.69 (1H, d, *J* 9.0, 14-H), 5.37 (1H, dd, *J* 11.0, 1.5, 20-*H*H), 5.20 (1H, dd, *J* 17.0, 1.5, 20-H*H*), 3.01 (1H, dd, *J* 11.0, 7.0, 11-H), 2.31 (1H, pent, *J* 7.0, 10-H), 2.27-2.22 (2H, m, 4-H, 8-*H*H), 2.16 (1H, dd, *J* 16.0, 9.0, 13-*H*H), 1.99 (3H, s, $COCH_3$), 1.69-1.58 (2H, m, 6-H, 7-*H*H), 1.55 (3H, s, 15-H_3), 1.37-1.30 (2H, m, 7-H*H*, 13-H*H*), 1.14 (3H, s, 18-H_3), 1.07 (3H, d, *J* 7.0, 17-H_3), 0.97 (1H, m, 8-H*H*), 0.8 (3H, d, *J* 7.0, 16-H_3). ESI-HRMS calc. for $C_{22}H_{32}O_4Na$ $[M+Na]^+$ 383.2198. Found 383.2193.

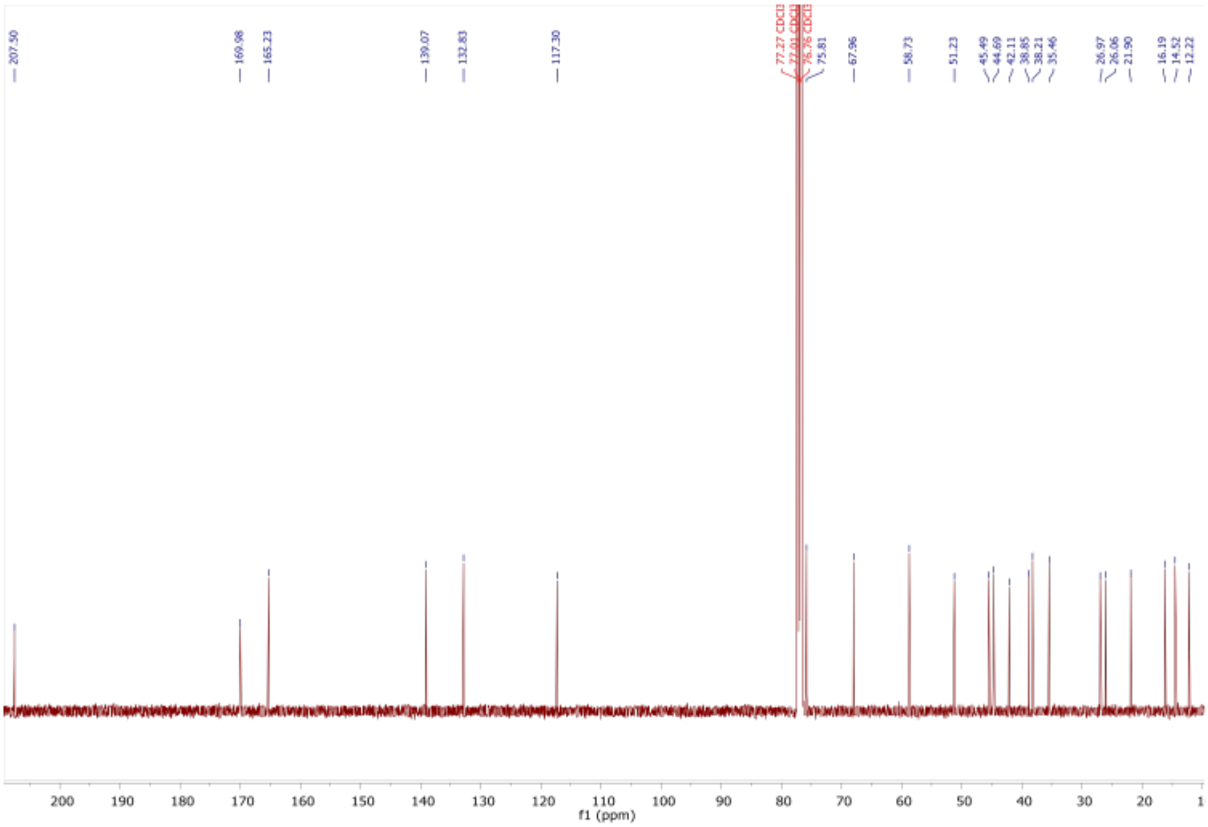

Supplementary Figure 49. ^{13}C-NMR spectrum of **20** in $CDCl_3$ (125 MHz). δ_C (125 MHz, $CDCl_3$) 207.5 (C-3), 170.0 (CO), 165.2 (C-1), 139.0 (C-19), 132.8 (C-2), 117.3 (C-20), 75.8 (C-11), 68.0 (C-14), 58.7 (C-4), 51.2 (C-9), 45.5 (C-13), 44.7 (C-12), 42.1 (C-5), 38.8 (C-8), 38.2 (C-6), 35.5 (C-10), 27.0 (C-7), 26.0 (C-18), 21.9 (CO*C*H_3), 16.2 (C-16), 14.5 (C-15), 12.2 (C-17). ESI-HRMS calc. for $C_{22}H_{32}O_4Na$ $[M+Na]^+$ 383.2198. Found 383.2193.

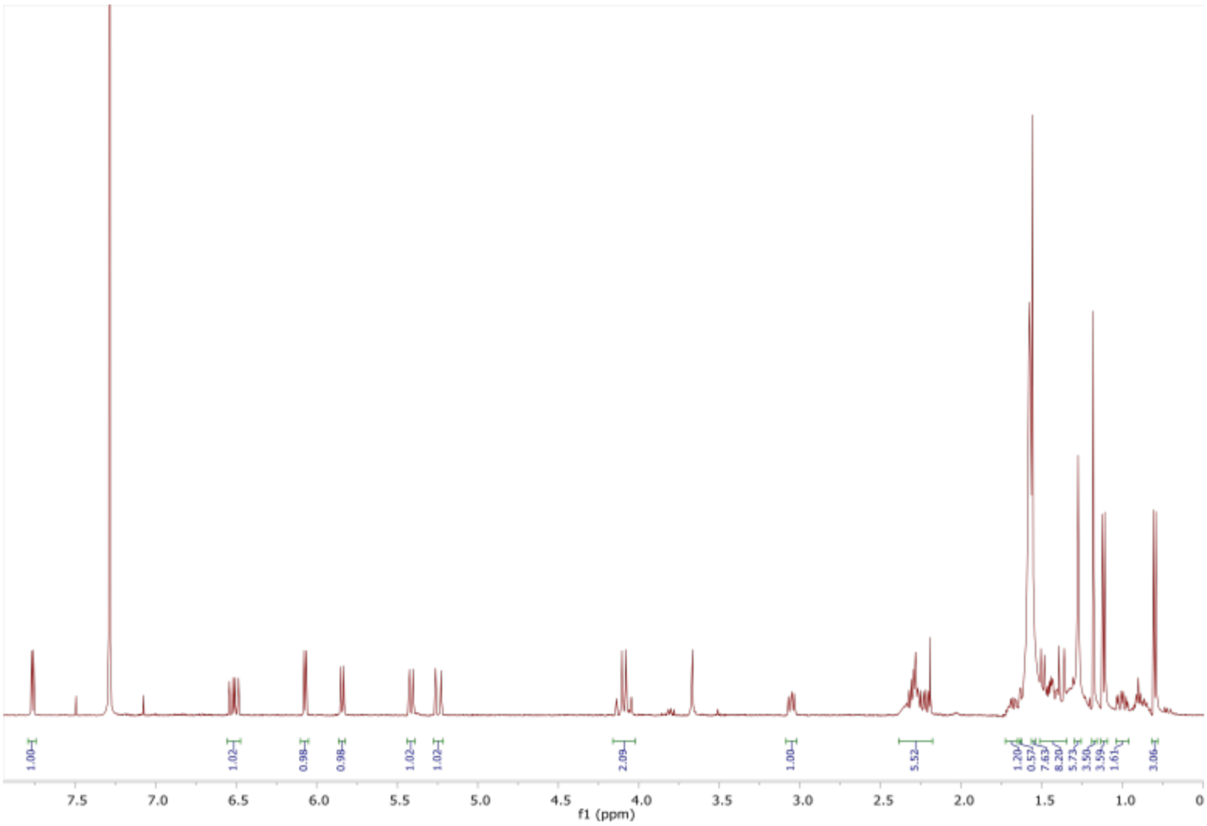

Supplementary Figure 50. ^{1}H-NMR spectrum of **21** in $CDCl_3$ (500 MHz). **21**. δ_H (500 MHz, $CDCl_3$) 7.74 (1H, d, *J* 6.0, 1-H), 6.49 (1H, dd, *J* 17.0, 11.0, 19-H), 6.05 (1H, d, *J* 6.0, 2-H), 5.82 (1H, d, *J* 9.0, 14-H), 5.39 (1H, dd, *J* 11.0, 1.5, 20-*H*H), 5.22 (1H, dd, *J* 11.0, 1.5, 20-H*H*), 4.10 and 4.04 (2H, 2 x d, AB, 14-OCH_2), 3.03 (1H, dd, *J* 11.0, 7.0, 11-H), 2.37-2.15 (4H, m, 10-H, 4-H, 8-*H*H, 13-*H*H), 1.71-1.32 (4H, m, 6-H, 7-H_2, 13-H*H*), 1.25 (3H, s, 15-H_3), 1.16 (3H, s, 18-H_3), 1.09 (3H, d, *J* 7.0, 17-H_3), 0.98 (1H, m, 8-H*H*), 0.78 (3H, d, *J* 7.0, 16-H_3). ESI-HRMS calc. for $C_{22}H_{32}O_5Na$ $[M+Na]^+$ 399.2161. Found 399.2142.

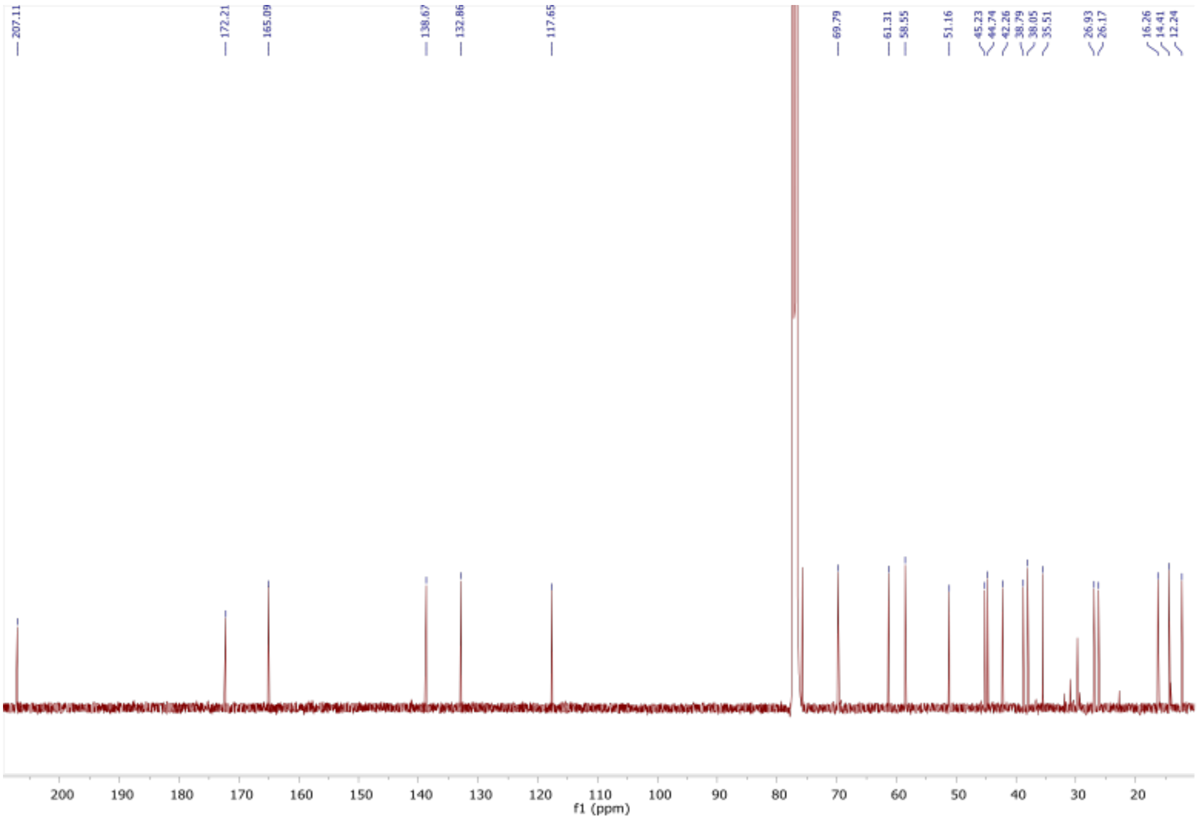

Supplementary Figure 51. ^{13}C-NMR spectrum of **21** in $CDCl_3$ (125 MHz). δ_C (100 MHz, $CDCl_3$) 207.1 (C-3), 172.2 (C-21), 165.1 (C-1), 138.7 (C-19), 132.9 (C-2), 117.6 (C-20), 75.8 (C-11), 69.8 (C-14), 61.3 (OCH_2), 58.5 (C-4), 51.2 (C-9), 45.2 (C-13), 44.7 (C-12), 42.3 (C-5), 38.8 (C-8), 38.1 (C-6), 35.5 (C-10), 26.9 (C-7), 26.2 (C-18), 16.3 (C-16), 14.4 (C-15), 12.2 (C-17). ESI-HRMS calc. for $C_{22}H_{32}O_5Na$ $[M+Na]^+$ 399.2161. Found 399.2142.

Supplementary Table 6. Plate-based antimicrobial bioassay on *B. subtilis*. For each of the five compounds tested (**1**, **6**, **21**, **20** and **18**), four different amounts were trialled (100, 25, 5 and 1 μg). Each experiment was carried out in triplicate, and the average size of the inhibition zone on the growth of the bacteria was calculated, with respective standard deviation.

Compound	Amount (μg)	Average inhibition zone (mm)	Standard deviation
**1**	100	12.8	0.8
	25	6.2	1.3
	5	3.7	0.3
	1	0.0	0.0
**6**	100	10.3	0.3
	25	5.8	0.6
	5	2.7	0.6
	1	0.0	0.0
**21**	100	15.7	1.8
	25	8.0	2.2
	5	2.5	0.9

	1	0.0	0.0
**20**	100	12.7	0.6
	25	8.2	0.6
	5	3.0	0.0
	1	0.0	0.0
**18**	100	8.0	0.0
	25	2.0	0.0
	5	0.0	0.0
	1	0.0	0.0

Supplementary Methods

Nucleic acids manipulation and construction of plasmids. Restriction digestions were carried out using restriction enzymes purchased from Fermentas or New England Biolabs, according to the manufacturer's protocols. PCR aimed at assessing presence of target DNA fragments was carried out using DreamTaq Green DNA Polymerase (Thermo Scientific) according to the manufacturer's protocol. High-fidelity PCR aimed at cloning DNA fragments for subsequent expression was carried out using Phusion High-fidelity DNA Polymerase (Thermo Scientific) according to the manufacturer's protocol and PCR products purified through GeneJET Gel Extraction Kit (Thermo Scientific). RT-qPCR was set up using SYBR Green PCR Master Mix (Thermo Scientific), according to the manufacturer's protocol, and performed with a Stratagene MX3005 qPCR system. RT-qPCR data were analysed with MXpro and Microsoft Excel software. Primers for PCR and RT-qPCR were purchased from Integrated DNA Technologies, and are listed in Supplementary Table 6. Integrity of genes after construction of plasmids was checked by sequencing (GATC Biotech). Plasmid DNA purification from *E. coli* was carried out using either GeneJET Plasmid Miniprep Kit (Thermo Scientific) or GeneJET Plasmid Midiprep Kit (Thermo Scientific). Plasmid purification from *S. cerevisiae* was done using Zymoprep Yeast Plasmid Miniprep I (Zymo Research) in order to rescue plasmids constructed through homologous recombination in yeast.

Cloning of intron-free genes and RT-qPCR. RNA purification was performed using E.Z.N.A. Plant RNA Kit (Omega Bio-Tek) according to the manufacturer's protocol for RNA purification from fungal samples. RNA purification was performed on *C. passeckerianus* CP1 in order to clone the intron-free genes of the pleuromutilin gene cluster to be used for heterologous expression, as well as on *C. passeckerianus* CP1 and CP-sil for the purpose of the RT-qPCR analysis. A plug of growing mycelium on a PDA plate was used to inoculate 100 mL of PVS medium (8 g L^{-1} vegetable oil, 35 g L^{-1} corn steep solid, 15 g L^{-1} glucose, 5 g L^{-1} calcium carbonate). After 5 days of growth at 25°C with shaking at 225 rpm, 1 mL of the fungal growth was used to inoculate 100 mL of CGC medium (50 g L^{-1} glucose, 5 g L^{-1} corn steep solid, 2 g L^{-1} calcium carbonate). After 5 days of incubation at 25°C with shaking at 225 rpm, the mycelium was harvested by centrifugation at 7,000 x *g* for 3 minutes and freeze-drying was used in order to dry the mycelium completely. E.Z.N.A. Plant RNA Kit (Omega Bio-Tek) was used to

perform RNA purification from 25 mg of dry mycelium and the purified RNA was visualised on a 1% agarose gel with ethidium bromide (0.5 μg mL^{-1}) to assess integrity of the nucleic acid. RNA was treated with RNase-free DNase I (Qiagen) in order to eliminate any trace of genomic DNA by adding 1 µL of DNase I and 1 µL of 10X reaction buffer with $MgCl_2$ to 1 µg of the purified RNA. DEPC treated water was added to the reaction mixture to make a final volume of 10 µL and the reaction was incubated at 37°C for 1 hour. The reaction was terminated by adding 1 µL of 0.5 M EDTA and incubating at 65°C for 10 minutes. First-Strand cDNA Synthesis Kit (Thermo Scientific) was used to perform first-strand cDNA synthesis and 1 μL of 100 μM Oligo$(dT)_{18}$ primer included in the kit was used to prime cDNA synthesis and selectively amplify from the mRNA. For the purpose of the cloning of the genes for consequent heterologous expression, high fidelity PCR amplification of the seven genes of the pleuromutilin cluster was carried out to obtain the full-length coding sequence and 10 bp of respectively 5' and 3' UTRs of each gene, using the primer sets listed in Supplementary Table 6. Specifically, amplification of *Pl-ggs* was done using primers pair Pl-ggs FF/Pl-ggs RR, *Pl-cyc* was amplified using primers pair Pl-cyc FF/Pl-cyc RR, *Pl-p450-1* with primers pair Pl-p450-1 FF/Pl-p450-1RR, *Pl-p450-2* with primers pair Pl-p450-2 FF/Pl-p450-2RR, *Pl-atf* with primers pair Pl-atf FF/Pl-atf RR, *Pl-sdr* with primers pair Pl-sdr FF/Pl-sdr RR. PCR products were cloned in pJET 1.2 cloning vectors and sequences of the genes were checked by sequencing (GATC Biotech) using the primers pJET1.2 FF and pJET1.2 RR. The gene sequence of the cloned product *Pl-cyc* was further checked by sequencing using two internal primers – Pl-cyc internal FF1 and Pl-cyc internal RR1 – as sequencing of the full-length gene could not be covered by using only the two external primers. For the purpose of the RT-qPCR, specific primer pairs (listed in Supplementary Table 6) were designed to amplify each of the genes of the pleuromutilin cluster, as well as the *beta-tubulin* gene (*Cp-beta-tub*) of *C. passeckerianus*, used as reference gene for the analysis. Specifically, *Cp-beta-tub* was amplified with primers pair Cp-beta-tub qPCR FF/ Cp-beta-tub qPCR RR, *Pl-ggs* with primers pair Pl-ggs qPCR FF/Pl-ggs qPCR RR, *Pl-cyc* was amplified using primers pair Pl-cyc qPCR FF/Pl-cyc qPCR RR, *Pl-p450-1* with primers pair Pl-p450-1 qPCR FF/Pl-p450-1 qPCR RR, *Pl-p450-2* with primers pair Pl-p450-2 qPCR FF/Pl-p450-2 qPCR RR, *Pl-atf* with primers pair Pl-atf qPCR FF/Pl-atf qPCR RR, *Pl-sdr* with primers pair Pl-sdr qPCR FF/Pl-sdr qPCR RR.

Supplementary Table 7. Primers sequences. Primers were designed based on the sequence data obtained from genome sequencing of *C. passeckerianus* DSMZ1602. * Primers used to amplify target genes before homologous recombination in yeast. The flanking sequences highlighted in red are homologous to the sequence of the plasmid backbone.

Primer	DNA Sequence (5'->3')	$T_{melting}$ (°C)	Description
Pl-p450-3 FF	CCCAACATCTATGGCTCCGTCAACG	61.5	

Pl-p450-3 RR	ACATGTGTATCTAGCCACTAGCAGG	58.0	Amplification of *Pl-p450-3* with UTRs
Pl-p450-3 START FF	ATGGCTCCGTCAACGGAACGTGCTC	65.1	Amplification of *Pl-p450-3*
Pl-p450-3 STOP RR	CTAGCCACTAGCAGGCTTCGTGAAC	61.1	
Padh-Pl-p450-3 FF*	TTTCTTTCAACACAAGATCCCAAAGT CAAAATGGCTCCGTCAACGGAACG	67.8	Amplification of *Pl-p450-3* to be used in yeast-based homologous recombination
Pl-p450-3-Teno RR*	GGTTGGCTGGTAGACGTCATATAATC ATACCTAGCCACTAGCAGGCTTCG	67.9	
Pl-p450-3 qPCR FF	GATCATGTACGGAACTTGGTC	53.1	Amplification of *Pl-p450-3* in RT-qPCR
Pl-p450-3 qPCR RR	GGAATGTGTTTCATCGCAGG	54.3	
Pl-atf FF	TGATACCACCATGAAGCCCTTCTCA	59.7	Amplification of *Pl-atf* with UTRs
Pl-atf RR	ACGGGCGTGCCTACTGTGCTACACG	66.6	
Pl-atf START FF	ATGAAGCCCTTCTCACCAGAACTTC	58.8	Amplification of *Pl-atf*
Pl-atf STOP RR	CTACTGTGCTACACGAGGGGGATTC	60.5	
Padh-Pl-atf FF*	TTTCTTTCAACACAAGATCCCAAAGT CAAAATGAAGCCCTTCTCACCAGA	66.7	Amplification of *Pl-atf* to be used in yeast-based homologous recombination
Pl-atf-TgpdA RR*	ACGACAATGTCCATATCATCAATCAT GACCCTACTGTGCTACACGAGGGG	68.1	
Pl-atf-Teno RR*	GGTTGGCTGGTAGACGTCATATAATC ATACCTACTGTGCTACACGAGGGG	67.7	
Pl-atf qPCR FF	TCAGGTTACTCAGAACCACAG	53.9	Amplification of *Pl-atf* in RT-qPCR
Pl-atf qPCR RR	TTAGAGCCAGACTTCAAGCC	54.5	
Pl-cyc FF	CTCTCATCTTCACTATGGGTCTATC	53.9	Amplification of *Pl-cyc* with UTRs
Pl-cyc RR	ACTAGCCATATCAATGGTGGATTCC	56.6	
Pl-cyc START FF	ATGGGTCTATCTGAAGATCTTCATG	54.0	Amplification of Pl-cyc
Pl-cyc STOP RR	TCAATGGTGGATTCCATTGCTCCCG	61.4	
Pl-cyc internal FF1	CCACTCACGACGCTGACATGAGCTC	63.1	Internal sequencing of *Pl-cyc*
Pl-cyc internal RR1	ACCTCGCTGAGGGTCGAGAACGACT	65.0	
Peno-Pl-cyc FF*	GTCGACTGACCAATTCCGCAGCTCGT CAAAATGGGTCTATCTGAAGATCT	68.3	Amplification of *Pl-cyc* to be used in yeast-based homologous recombination
Pl-cyc-Teno RR*	GGTTGGCTGGTAGACGTCATATAATC ATACTCAATGGTGGATTCCATTGC	66.3	
Pl-cyc qPCR FF	TACCTACTTCCGTTGCTACT	52.5	Amplification of *Pl-cyc* in RT-qPCR
Pl-cyc qPCR RR	GAGTACCATTCTGATACATTCC	50.4	
Pl-ggs FF	AATTCATACGATGAGAATACCTAAC	50.6	Amplification of *Pl-ggs* with UTRs
Pl-ggs RR	AGATTCTTATCTACTCTGCGATGTA	52.9	
Pl-ggs START FF	ATGAGAATACCTAACGTCTTTCTCT	53.3	Amplification of *Pl-ggs*
Pl-ggs STOP RR	CTACTCTGCGATGTACAACTTTTCC	55.6	
Padh-Pl-ggs FF*	TTTCTTTCAACACAAGATCCCAAAGT CAAAATGAGAATACCTAACGTCTT	64.1	Amplification of *Pl-ggs* to be used in yeast-based homologous recombination
Pl-ggs-TgpdA RR*	ACGACAATGTCCATATCATCAATCAT GACCCTACTCTGCGATGTACAACT	66.5	
Pl-ggs qPCR FF	ATCTGGTGGCGTGACCAGCG	62.7	Amplification of *Pl-ggs* in RT-qPCR
Pl-ggs qPCR RR	AGTAGACACCAATACGGAGC	53.9	
Pl-p450-1 FF	TATTCACGCAATGCTGTCCGTCGAC	61.2	Amplification of *Pl-p450-1* with UTRs
Pl-p450-1 RR	TGTTAGGAGGCTACAACGCAGCGAA	62.2	
Pl-p450-1 START FF	ATGCTGTCCGTCGACCTCCCGTCTG	66.0	Amplification of *Pl-p450-1*
Pl-p450-1 STOP RR	CTACAACGCAGCGAACGCTTCCTTA	61.2	
Padh-Pl-p450-1 FF*	TTTCTTTCAACACAAGATCCCAAAGT CAAAATGCTGTCCGTCGACCTCCC	68.3	Amplification of *Pl-p450-1* to be used in yeast-based homologous recombination
Pl-p450-1-TgpdA RR*	ACGACAATGTCCATATCATCAATCAT GACCCTACAACGCAGCGAACGCTT	68.7	
Pl-p450-1-Teno RR*	GGTTGGCTGGTAGACGTCATATAATC ATACCTACAACGCAGCGAACGCTT	68.4	
Pl-p450-1 qPCR FF	ATCAACTGGTCTCTTCATCA	50.8	Amplification of *Pl-p450-1* in RT-qPCR
Pl-p450-1 qPCR RR	GAACGCTTCCTTAATCAAGTC	51.4	

Pl-p450-2 FF	TCGGACGACTATGAATCTTTCTGCT	57.6	Amplification of *Pl-p450-2* with UTRs
Pl-p450-2 RR	ATCCCCCTATCTAATAGTCTGCAAC	55.7	
Pl-p450-2 START FF	ATGAATCTTTCTGCTCTGAAGGCTG	57.2	Amplification of *Pl-p450-2*
Pl-p450-2 STOP RR	CTAATAGTCTGCAACATCGTGGATC	55.3	
Peno-Pl-p450-2 FF*	GTCGACTGACCAATTCCGCAGCTCGT CAAAATGAATCTTTCTGCTCTGAA	68.1	Amplification of *Pl-p450-2* to be used in yeast-based homologous recombination
Pl-p450-2-Teno RR*	GGTTGGCTGGTAGACGTCATATAATC ATACCTAATAGTCTGCAACATCGT	65.5	
Pl-p450-2 qPCR FF	CCTCCTACAAATCCGACGAA	54.2	Amplification of *Pl-p450-2* in RT-qPCR
Pl-p450-2 qPCR RR	ACGAGATGCCTAAAGAATTCCT	53.8	
Pl-sdr FF	AGCAGTGACCATGGAAGGCAAGGTC	63.1	Amplification of *Pl-sdr* with UTRs
Pl-sdr RR	ATACGGCGACCTAAATGACACTCCA	59.7	
Pl-sdr START FF	ATGGAAGGCAAGGTCGCAATCGTCA	62.8	Amplification of *Pl-sdr*
Pl-sdr STOP RR	CTAAATGACACTCCACCCGTTATCG	57.2	
Peno-Pl-sdr FF*	GTCGACTGACCAATTCCGCAGCTCGT CAAAATGGAAGGCAAGGTCGCAAT	70.8	Amplification of *Pl-sdr* to be used in yeast-based homologous recombination
Pl-sdr-Teno RR*	GGTTGGCTGGTAGACGTCATATAATC ATACCTAAATGACACTCCACCCGT	67.2	
Padh-Pl-sdr FF*	TTTCTTTCAACACAAGATCCCAAAGT CAAAATGGAAGGCAAGGTCGCAAT	67.3	
Pl-sdr qPCR FF	GAGGTATTGAAGGTTATGAAGGTC	53.0	Amplification of *Pl-sdr* in RT-qPCR
Pl-sdr qPCR RR	CATCGTCCTTGAACATCCAC	53.5	
pJET1.2 FF	CGACTCACTATAGGGAGAGCGGC	60.6	Sequencing of inserts from pJET1.2
pJET1.2 RR	AAGAACATCGATTTTCCATGGCAG	56.0	
beta-tub RIB40 FF	CCAAGAACATGATGGCTGCT	55.7	Amplification and qPCR of *beta-tubulin* from *A. oryzae*
beta-tub RIB40 RR	CTTGAAGAGCTCCTGGATGG	55.1	

Homologous recombination in *S. cerevisiae*. Homologous recombination in yeast (*S. cerevisiae*) was used to construct expression vectors according to the procedure described by Ma, *et al*[5]. The expression vectors for transformation of *A. oryzae* were constructed using as backbones the plasmids pTYGSarg, pTYGSade, pTYGSbar provided by Dr Colin Lazarus. These are derivatives of pTAYAGSarg3P[6] and have been made by adding the respective terminators for the provided promoters; each one has a different selectable marker for transformation of *A. oryzae* (Supplementary Table 7). High fidelity PCR was used to amplify the genes of the pleuromutilin cluster from their cloning vectors pJET1.2 and amplicons were purified through GeneJET Gel Extraction Kit (Thermo Scientific). The plasmid backbone was linearised by restriction digestion with *Asc*I (30 units). Competent cells of *S. cerevisiae* BY4742 were prepared and transformed according to the lithium acetate/single-stranded carrier DNA/polyethylene glycol method described by Gietz and Woods[7]. A 10 mL YPD (10 g L^{-1} yeast extract, 20 g L^{-1} bactopeptone, 20 g L^{-1} D-glucose) culture was set up using a single colony of *S. cerevisiae* taken from a fresh plate of YPAD (same as YPD with 20 g L^{-1} agar) and shaken at 225 rpm at 28°C overnight. This starter culture was used to inoculate 40 mL of YPD and shaken at 225 rpm at 28°C for five hours. The yeast culture was centrifuged and pelleted cells washed twice in sterile water, then resuspended in 1 mL of 0.1 M LiOAc solution. Cells were pelleted again, resuspended in 400 μL of 0.1 M LiOAc solution and 50 μL aliquots transferred to new tubes. Each tube was added with 240 μL of 50% PEG 3350 solution, 36 μL of 1 M LiOAc solution, 50 μL of salmon sperm single-stranded

DNA and 34 μL of DNA fragments (typically 5 μL of linearised backbone plasmid and of each PCR product, then making up to final volume with sterile water). Cells were incubated at 30°C for 30 minutes, then at 42°C for 30 minutes. Pelleted cells were resuspended in 500 μL of sterile water and 200 μL of each transformation mixture were spread on a plate of synthetic medium SM lacking uracil (1.7 g L^{-1} yeast nitrogen base without amino acids, 5 g L^{-1} ammonium sulphate, 20 g L^{-1} D-glucose, 0.77 g L^{-1} Yeast Synthetic Drop-out Medium Supplements without uracil, 20 g L^{-1} agar) then incubated at 28°C for 2-3 days. The resultant colonies were pooled together and plasmid DNA was purified using Zymoprep Yeast Plasmid Miniprep I (Zymo Research). The rescued plasmid DNA was transformed into *E. coli* One Shot® ccdB Survival™ 2 $T1^R$ competent cells (Life Technologies) for plasmid propagation following standard procedures, then checked by restriction digestion, PCR amplification of the genes inserted and DNA sequencing (GATC Biotech).

Supplementary Table 8. List of backbone plasmids used to construct expression vectors with respective promoters and terminators. Selectable markers. *ArgB*: *A. nidulans* ornithine carbamoyltransferase (OCTase) gene; *AdeA*: *A. oryzae* gene homolog of the phosphoribosylaminoimidazolesuccinocarboxamide synthase gene *ADE1* of *S. cerevisiae*, which encodes an enzyme involved in purine biosynthesis; *Bar*: *Streptomyces* spp. phosphinothricin acetyltransferase gene. Promoters and Terminators. *Padh*/*Tadh*: *A. oryzae* alcohol dehydrogenase promoter/terminator; *PgpdA*/*TgpdA*: *A. nidulans* Glyceraldehyde 3'-phosphate dehydrogenase promoter/terminator; *Peno*/*Teno*: *A. oryzae* enolase promoter/terminator.

Backbone vector	Selectable marker gene	Selection based on	Promoter	Terminator
**pTYGSarg**	*argB*	Complementation of auxotrophy for arginine biosynthesis	Padh	Tadh
			PgpdA	TgpdA
			Peno	Teno
**pTYGSade**	*adeA*	Complementation of auxotrophy for adenine biosynthesis	Padh	Tadh
			PgpdA	TgpdA
			Peno	Teno
**pTYGSbar**	*bar*	Resistance to the herbicide Basta	Padh	Tadh
			PgpdA	TgpdA
			Peno	Teno

Construction of plasmids for expression in *A. oryzae*. Expression vectors were constructed to express the genes of the pleuromutilin gene cluster from *C. passeckerianus* in *A. oryzae*. Two μg of the chosen backbone plasmid pTYGSarg, pTYGSade and pTYGSbar were linearised through restriction digestion with *Asc*I (30 units) by incubating at 37°C for 3 hours. The genes to insert in the vectors were amplified from their respective pJET 1.2 plasmids using High Fidelity PCR with 30 bp-extended primers to create an overlap with the sequence of the promoter and terminator of the plasmid, then were joined to the chosen linearised plasmid through homologous recombination in yeast as described previously. Specifically, to construct pTYGSargGGSCyc the backbone plasmid pTYGSarg was linearised and recombined with the *Pl-ggs* and *Pl-cyc* genes – respectively amplified using the primers pairs Padh-Pl-

ggs FF/Pl-ggs-TgpdA RR and Peno-Pl-cyc FF/Pl-cyc-Teno RR. To build pTYGSadeP1 the plasmid backbone pTYGSade was linearised and recombined with *Pl-p450-1*, previously amplified using the primers pair Padh-Pl-p450-1 FF/Pl-p450-1-Teno RR. To build pTYGSadeP1P2 the plasmid backbone pTYGSade was linearised and recombined with *Pl-p450-1* and *Pl-p450-2*, previously amplified using the primers pairs Padh-Pl-p450-1 FF/Pl-p450-1-Tgpd RR and Peno-Pl-p450-2 FF/Pl-p450-2-Teno RR. The vector pTYGSbarATFSDR was constructed by joining the linearised plasmid pTYGSbar with the genes *Pl-atf* and *Pl-sdr*, respectively amplified using the primers pairs Padh-Pl-atf FF/Pl-atf-TgpdA RR and Peno-Pl-sdr FF/Pl-sdr-Teno RR. The vector pTYGSbarSDR was built recombining the linearised backbone pTYGSbar with the gene *Pl-sdr*, previously amplified using the primers pair Padh-Pl-sdr FF/Pl-sdr-Teno RR. The vector pTYGSbarATF was built recombining the linearised backbone pTYGSbar with the gene *Pl-atf*, previously amplified using the primers pair Padh-Pl-atf FF/Pl-atf-Teno RR. Transformant yeast colonies were selected for restored prototrophy for uracil, conferred by the selectable marker gene *URA3* contained in the backbone plasmids. Plasmids were rescued from yeast, shuttled in *E. coli* for propagation and checked through restriction digestion, PCR amplification of the genes inserted and DNA sequencing (GATC Biotech). See Supplementary Table 8 for a summary of the expression vectors built, and Supplementary Figure 45 for plasmid maps.

Supplementary Table 9. Summary of expression vectors built with the genes of the pleuromutilin gene cluster from *C. passeckerianus*.

Expression vector	Genes
pTYGSargGGSCyc	*Pl-ggs*
	Pl-cyc
pTYGSadeP1	*Pl-p450-1*
pTYGSadeP1P2	*Pl-p450-1*
	Pl-p450-2
pTYGSadeP3	*Pl-p450-3*
pTYGSbarATFSDR	*Pl-atf*
	Pl-sdr
pTYGSbarSDR	*Pl-sdr*
pTYGSbarATF	*Pl-atf*

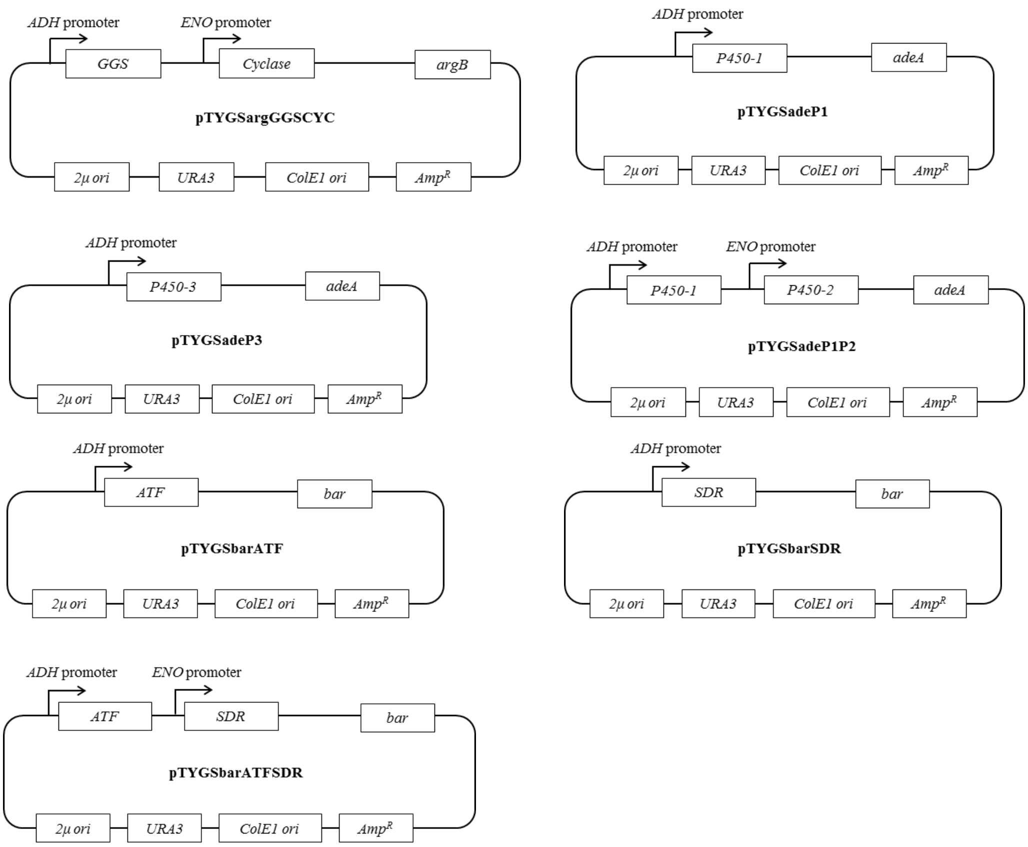

Supplementary Figure 52. Plasmid maps of the expression vectors built in this work.

Antimicrobial bioassay. *Bacillus subtilis* was used to test antibiotic activity of compounds in plate-based bioassays. Briefly, *B. subtilis* spores were prepared by inoculating 50 mL of TSB (30 g L^{-1} tryptic soy broth premix) and grown overnight at 30°C. One mL of the culture was used to inoculate 100 mL of TSA (30 g L^{-1} tryptic soy broth premix, 5 g L^{-1} agar) and spread over the surface of the medium and incubated at 30°C for 6 days. Sterile distilled water was used to harvest the culture, spores were activated (70°C for 30 minutes), then titrated and diluted to a concentration of $1x10^{-9}$ spores mL^{-1}. The spores were then aliquoted and stored at 4°C until needed. Bioassays were used to test the antibiotic activity of compounds **20** and **21**, in comparison to that of **1**, **6** and **18**. 5 μL of *B. subtilis* spores and 37.5 μL of 4 % TTC (2,3,5-Triphenyl-2H-tetrazolium chloride) were poured onto the plate. A sterile round piece of filter paper was placed in the centre of the plate, and an appropriate amount of compound to be tested was pipetted over it. Plates were then incubated at 30°C for 24-48 hours, after which colony growth and inhibition zones were measured. Each strain was tested in triplicate and the standard deviation was calculated.

Protoplast-mediated transformation of *A. oryzae*. Protoplast-mediated transformation of *A. oryzae* was undertaken in order to insert the genes of the pleuromutilin gene cluster in different combination and consequently analysing their metabolome. The protocol described by Halo, *et al.*[8] was followed. In principle, spores from *A. oryzae* were collected in sterile water from a ten-day old plate, conidia were

separated from hyphal debris by filtration through sterile Miracloth® and used to inoculate 50 mL of GN (10 g L^{-1} D-glucose, 20 g L^{-1} nutrient broth no.2 (LabM)). Conidia were incubated overnight at 28°C with shaking at 225 rpm and then centrifuged at 8,000 x *g* for 10 minutes. Pelleted conidia were resuspended in 10 mL of protoplasting solution (20 g L^{-1} lysing enzyme from *Trichoderma harzianum*, 10 g L^{-1} Driselase from *Basidyomecetes* sp., 0.8 M NaCl) and incubated in gentle mixing at room temperature for 1 hour. Protoplasts were separated from hyphal debris by filtration through sterile Miracloth®, centrifuged at 3,000 x *g* for 5 minutes and resuspended in Solution 1 (0.8 M NaCl, 10 mM $CaCl_2$, 50 mM Tris-HCl pH 7.5) to a final concentration of $1x10^7$ mL^{-1}. Aliquots of 100 µL of protoplasts were added with 10 µg of plasmid DNA and incubated on ice for 2 minutes. One mL of Solution 2 (600 g L^{-1} PEG 3,350, 10 mM $CaCl_2$, and 50 mM Tris-HCl pH 7.5) was added and the mixture incubated at room temperature for 20 minutes. Plates were prepared to plate out the transformation mixture. When the selectable marker *argB* was exploited to transform *A. oryzae* NSAR1, then CZDS base plates selective for arginine (35 g L^{-1} Czapek-dox broth, 1 M sorbitol, 1.5 g L^{-1} methionine, 0.1 g L^{-1} adenine, 2 g L^{-1} ammonium sulphate, 15 g L^{-1} agar) were used. When the *adeA* selectable marker was exploited to further transform the arginine-prototroph *A. oryzae* GC strain, then CZDS base plates selective for adenine and arginine (35 g L^{-1} Czapek-dox broth, 1 M sorbitol, 1.5 g L^{-1} methionine, 2 g L^{-1} ammonium sulphate, 15 g L^{-1} agar) were used. Likewise, the same medium, CZDS base selective for adenine and arginine, was also used when the arginine-prototroph *A. oryzae* GC strain was transformed with a combination of plasmids containing *adeA* and *bar* selectable markers. When *A. oryzae* NSAR1 was transformed with a combination of expression vectors containing *adeA* and *bar* selectable markers, then CZDS base plates selective for adenine (35 g L^{-1} Czapek-dox broth, 1 M sorbitol, 1.5 g L^{-1} arginine, 1.5 g L^{-1} methionine, 2 g L^{-1} ammonium sulphate, 15 g L^{-1} agar) were used.Five mL of the appropriate melted selective CZDS top medium (same composition as the respective CZDS but with 8 g L^{-1} agar instead of 15 g L^{-1} agar) were added to the transformation mixture and everything was poured over the prepared 15 mL CZDS base plate. Transformation plates were incubated at 28°C for 2-3 days. Transformant colonies were subcultured at least three times streaking to single colony on a plate of the appropriate selective medium before proceeding with further analysis.

Generation of *A. oryzae* transformant strains. Firstly, *A. oryzae* NSAR1 was transformed with pTYGSargGGSCyc, exploiting the *argB* selectable marker. This achieved production of the transformant strain GC (containing *Pl-ggs* and *Pl-cyc* from *C. passeckerianus*). PCR amplification was used to screen for presence of the transgenes inserted; amplification of *Pl-ggs* was achieved using primers pair Pl-ggs START FF/Pl-ggs STOP RR, *Pl-cyc* was amplified using primers pair Pl-cyc START FF/Pl-cyc STOP RR. The arginine-prototroph strain GC was further transformed in independent events with expression vectors pTYGSadeP1 and pTYGSadeP1P2, by exploiting the *adeA* selectable marker. These transformation events achieved generation of transformant strains GCP1 (containing *Pl-ggs*, *Pl-cyc* and *Pl-p450-1* from *C. passeckerianus*), and GCP1P2 (containing *Pl-ggs*,

Pl-cyc, *Pl-p450-1* and *Pl-p450-2* from *C. passeckerianus*). PCR amplification with primers pair Pl-p450-1 START FF/Pl-p450-1 STOP RR was used to test for insertion of *Pl-p450-1* in GCP1 and GCP1P2, whereas primers pair Pl-p450-2 START FF/Pl-p450-2 STOP RR was used to test for presence of *Pl-p450-2* in strain GCP1P2. A combination of pTYGSadeP1P2 (2 μl) and pTYGSbarSDR (8 μl) was introduced into GC, exploiting the selectable marker *adeA*, to produce strain GCP1P2S (containing *Pl-ggs*, *Pl-cyc*, *Pl-p450-1*, *Pl-p450-2* and *Pl-sdr* from *C. passeckerianus*). Presence of the transgenes inserted was assessed with PCR amplification using primers pairs Pl-p450-1 START FF/Pl-p450-1 STOP RR for *Pl-p450-1*, Pl-p450-2 START FF/Pl-p450-2 STOP RR for *Pl-p450-2*, and Pl-sdr START FF/Pl-sdr STOP RR for *Pl-sdr*. A combination of pTYGSadeP1P2 and pTYGSbarATFSDR was used to transform GC, exploiting the selectable marker *adeA*, to achieve generation of strain GCP1P2AS (containing *Pl-ggs*, *Pl-cyc*, *Pl-p450-1*, *Pl-p450-2*, *Pl-atf* and *Pl-sdr* from *C. passeckerianus*). Presence of the transgenes inserted was assessed with PCR amplification using primers pairs Pl-p450-1 START FF/Pl-p450-1 STOP RR for *Pl-p450-1*, Pl-p450-2 START FF/Pl-p450-2 STOP RR for *Pl-p450-2*, Pl-atf START FF/Pl-atf STOP RR for *Pl-atf*, and Pl-sdr START FF/Pl-sdr STOP RR for *Pl-sdr*. *A. oryzae* transformant strains to be employed in feeding experiments for conversion of **4** and **5** into **1**, were generated by transforming *A. oryzae* NSAR1 and exploiting the *adeA* selectable marker. Specifically, strain AP3 (containing *Pl-p450-3* and *Pl-atf* from *C. passeckerianus*) was generated by transforming *A. oryzae* NSAR1 with a combination of pTYGSadeP3 (2 μl) and pTYGSbarATF (8 μl). Insertion of the transgenes was confirmed with PCR amplification using primers pairs Pl-p450-3 START FF/Pl-p450-3 STOP RR for *Pl-p450-3* and Pl-atf START FF/Pl-atf STOP RR for *Pl-atf*. Strain SAP3 was obtained by transforming *A. oryzae* NSAR1 with a combination of pTYGSadeP3 (2 μl) and pTYGSbarATFSDR (8 μl). Insertion of the transgenes was confirmed with PCR amplification with primers pairs Pl-p450-3 START FF/Pl-p450-3 STOP RR for *Pl-p450-3*, Pl-atf START FF/Pl-atf STOP RR for *Pl-atf*, and Pl-sdr START FF/Pl-sdr STOP RR for *Pl-sdr*.

Ethyl acetate extractions of metabolites. Extractions with ethyl acetate were performed on *A. oryzae* NSAR1 and *A. oryzae* transformant strains in order to analyse their metabolome through analytical HPLC-MS. Spores from growing plates were collected in sterile water and used to inoculate 100 mL of CMP medium (35 g L^{-1} Czapek Dox liquid, 20 g L^{-1} maltose, 10 g L^{-1} peptone) in a 250-mL flask, then grown at 28°C with shaking at 225 rpm for ten days. Each mycelium was homogenised, pH was reduced to a value of 3 units with concentrated HCl, and an equal volume of ethyl acetate was added. A separation funnel was used to separate the organic and water phase. The organic phase was collected. The water phase was extracted again with an equal volume of ethyl acetate as described above, which was pooled with the previously collected one. This was dried with anhydrous $MgSO_4$ (10 g L^{-1}), which was removed by filtration. The organic phase was then concentrated using a rotary evaporator over a water bath warmed at 37°C, with rotation of the flask of 100 rpm. Dried crude extract was resuspended in acetonitrile (10 mg mL^{-1}) and transferred to an HPLC vial. The same procedure was adopted when

wanting to purify compounds through preparative HPLC-MS. In this case the fungal culture was scaled-up to 1 L in CMP medium instead of 100 ml, then the same protocol as for ethyl acetate extractions for analytical HPLC-MS was followed.

TLC. One-direction thin-layer chromatography (TLC) was used to separate and purify compounds from strains GC, AP3 and SAP3 after chemical extractions. For analytical TLC, fungal crude extract was transferred to a 2x10 cm TLC plate (TLC silica gel 60 F_{254}; Merck, Darmstadt, Germany) and plate developed in petroleum spirit-ethyl acetate (9:1) for NSAR1 GC and petroleum spirit-ethyl acetate (1:1) for strains AP3 and SAP3. The plate was dried and visualised after submerging in potassium permanganate solution (1.5 g of $KMnO_4$, 10 g K_2CO_3 and 1.25 mL 10% NaOH in 200 mL of water). Unsaturated compounds were shown as yellow spots, and R_f value recorded. For purification of such compounds preparative TLC was undertaken, using a 20x20 cm TLC plate (TLC silica gel 60 F254; Merck, Darmstadt, Germany), developed in petroleum spirit-ethyl acetate (9:1) for GC and petroleum spirit-ethyl acetate (1:1) for strains AP3 and SAP3. The plate was dried, a 1 cm-strip was cut from one side, and submerged in potassium permanganate solution. A spatula was used to scrape the silica off the plate in correspondence to the visualised spots developed in the strip with potassium permanganate solution. The purified compounds were extracted with methanol from the silica, collected in a pre-weighed glass vial with screw cap and dried under a flow of dry N_2 gas.

Analytical HPLC-MS. Analytical HPLC-MS was performed using a Waters 2767 HPLC system with a Waters 2545 pump system, and a Phenomenex LUNA column (2.6 μ, C_{18}, 100 Å, 4.6 × 100 mm) equipped with a Phenomenex Security Guard precolumn (Luna C_5 300 Å) for reverse-phase chromatography. UV absorbance was detected between 200 nm and 400 nm with a Waters 2998 diode array detector, with simultaneous electrospray mass spectrometry detection in positive (ES^+) and negative (ES^-) mode with a Waters Quattro-Micro spectrometer, detecting an *m/z* (mass/charge) range between 150 and 800 Da. A Waters 2424 Evaporative Light Scattering Detector Chromatography (ELSD) was also coupled to the HPLC-MS and was used to measure the amount of light scattered by the particles of solute contained in the samples, which ultimately gave an estimate of the concentration of material eluting. For the reverse-phase chromatography a gradient of solvents was used (A, HPLC grade H_2O containing 0.05% formic acid; B, HPLC grade CH_3CN containing 0.045% formic acid) with the following program: 0 minutes, 10% B; 15 minutes, 90% B; 16 minutes 95% B; 17 minutes 95% B; 18 minutes 10% B, 20 minutes 10% B. Flow rate was set at 1 mL min^{-1}. The data were analysed using Waters MassLynx™ V4.1 Software.

Preparative HPLC-MS. Preparative HPLC-MS was used in order to purify target compounds from the crude extract of transformant *A. oryzae* strains. For this purpose, the crude extract obtained from a 1 L culture of the fungal strain was used. Preparative HPLC-MS was achieved through a Waters mass-directed autopurification system with a Waters 2767 autosampler and Waters 2545 pump system, a

Phenomenex LUNA column (5 μ, C_{18}, 100 Å, 10 × 250 mm) for reverse-phase chromatography, equipped with a Phenomenex Security Guard precolumn (Luna C_5 300 Å) eluted at 16 mL min^{-1}. UV absorbance was detected between 200 and 400 nm with a Waters 2998 diode array detector, mass spectrometry with a Waters Quattro Micro, and approximate amount of compound eluted was evaluated with Waters 2424 for ELSD. For the reverse-phase chromatography a gradient of solvents was used (A, HPLC grade H_2O containing 0.05% formic acid; B, HPLC grade CH_3CN containing 0.045% formic acid) with the following program: 0 minutes, 5% B; 2 minutes, 10% B; 20 minutes, 90% B; 21 minutes 95% B; 26 minutes 95% B; 27 minutes 5% B, 30 minutes 5% B. Specific compounds were collected in glass tubes, fractions for the same compound were pooled in a pre-weighed glass vial with screw cap and dried under a flow of dry N_2 gas.

NMR spectroscopy. Nuclear magnetic resonance spectroscopy (NMR Spectroscopy) was used to characterise the metabolites produced and purified from *A. oryzae* transformant strains. After purification, dried compounds were dissolved in either $CDCl_3$ or CD_3OD, and NMR analyses were conducted on Agilent VNMRS500 or Agilent V400-MR spectrometer, ^{1}H-NMR at either 500 or 400 MHz and ^{13}C-NMR at 125 MHz. Coupling constant (*J*) was expressed in Hz. Chemical shifts were recorded in parts per million (ppm) and expressed relative to either the proton resonance δ 7.26 of $CHCl_3$ or δ 4.78 of CH_3OH for ^{1}H-NMR, and δ 77.16 of $CDCl_3$ for ^{13}C-NMR.

Supplementary References

1. Hasler, H. Neue Aspekte der Biosynthese von Pleuromutilin. Ph.D. thesis deposited at ETH Zurich (1979).
2. Schulz, G. & Berner, H. Chemie der pleuromutiline-VI : Vergleichende untersuchung der ^{13}C-NMR spektren des tricyclischen diterpens mutilin und einer reihe von mutilinderivaten. *Tetrahedron* **40**, 905-917 (1984).
3. Fazakerley, N.J., Helm, M.D. & Procter, D.J. Total Synthesis of (+)-Pleuromutilin. *Chemistry – A European Journal* **19**, 6718-6723 (2013).
4. Wang, H, Andemichael, Y.W., Vogt, F.G. A Scalable Synthesis of 2S-Hydroxymutilin via a Modified Rubottom Oxidation. *The Journal of Organic Chemistry* **74**, 478-481 (2009).
5. Ma, H., Kunes, S., Schatz, P. J.; Botstein, D. Plasmid construction by homologous recombination in yeast. *Gene* **58**, 201-2016 (1987).
6. Pahirulzaman, K.A.K., Williams, K. & Lazarus, C.M. A toolkit for heterologous expression of metabolic pathways in *Aspergillus oryzae*. *Methods in Enzymology* **517**, 241-260 (2012).
7. Gietz, D.R. & Woods, R.A. Transformation of yeast by lithium acetate/single-stranded carrier DNA/polyethylene glycol method. *Methods in Enzymology* **350**, 87-96 (2002).
8. Halo, L.M. *et al*. Late Stage Oxidations during the Biosynthesis of the 2-Pyridone Tenellin in the Entomopathogenic Fungus *Beauveria bassiana*. *Journal of the American Chemical Society* **130**, 17988-17996 (2008).
